# Yield-related salinity tolerance traits identified in a nested association mapping (NAM) population of wild barley

**DOI:** 10.1038/srep32586

**Published:** 2016-09-02

**Authors:** Stephanie Saade, Andreas Maurer, Mohammed Shahid, Helena Oakey, Sandra M. Schmöckel, Sónia Negrão, Klaus Pillen, Mark Tester

**Affiliations:** 1 King Abdullah University of Science and Technology (KAUST), Biological and Environmental Sciences and Engineering (BESE), Thuwal, 23955-6900, Saudi Arabia; 2 Institute of Agricultural and Nutritional Sciences, Martin Luther University Halle-Wittenberg, Betty-Heimann-Str. 3, 06120 Halle, Germany; 3 International Center for Biosaline Agriculture (ICBA), Dubai, United Arab Emirates

## Abstract

Producing sufficient food for nine billion people by 2050 will be constrained by soil salinity, especially in irrigated systems. To improve crop yield, greater understanding of the genetic control of traits contributing to salinity tolerance in the field is needed. Here, we exploit natural variation in exotic germplasm by taking a genome-wide association approach to a new nested association mapping population of barley called HEB-25. The large population (1,336 genotypes) allowed cross-validation of loci, which, along with two years of phenotypic data collected from plants irrigated with fresh and saline water, improved statistical power. We dissect the genetic architecture of flowering time under high salinity and we present genes putatively affecting this trait and salinity tolerance. In addition, we identify a locus on chromosome 2H where, under saline conditions, lines homozygous for the wild allele yielded 30% more than did lines homozygous for the Barke allele. Introgressing this wild allele into elite cultivars could markedly improve yield under saline conditions.

Over one billion hectares of the world’s land are affected by soil salinity, a major constraint on agricultural production^1^. Salinity tolerance in plants is under complex polygenic control and several genes have been proposed to be involved with salinity-tolerance traits^2^. However, what matters to the farmer and brings economic benefit to the agricultural sector is a plant’s tolerance to high salinity under field conditions during the reproductive stage.

In addition to its economic importance, barley is a suitable model for studying salinity tolerance in plants because it is the most salt tolerant of the cereal crops^3^. In this study, we used a nested association mapping (NAM) population of barley called “HEB-25”. HEB-25 was developed using wild barley (*Hordeum vulgare* ssp*. spontaneum*) donors from the Fertile Crescent^4^ that are likely adapted to high salinity and thus are likely to harbor salinity-tolerance alleles that were not eroded during domestication. At the same time, approximately 72% of HEB-25’s genome comes from the German elite cultivar Barke^4^, rendering it capable of reaching the reproductive stage and producing high yields in the field.

Here, we present a genome-wide association study (GWAS) in the wild barley NAM population HEB-25. This population, comprising 1,336 individuals belonging to 25 families (Supplementary Table 1), was evaluated for ten traits related to agronomic performance and yield in the field for two consecutive years (Supplementary Table 2) to identify loci associated with salinity tolerance using single nucleotide polymorphism (SNP) information. Plants were stressed with salt via drip irrigation in the sandy soil and dry environment of our field site, located at the International Center for Biosaline Agriculture (ICBA) in Dubai, UAE. This controlled, yet field-based, environment has for the first time, enabled the quantification of the effects of salinity on a large number of yield-related traits such as flowering time and the harvest index in a large mapping population. We dissect the genetic architecture of flowering time under high salinity and suggest putative genes underlying the observed quantitative trait loci (QTL). In addition to the loci identified previously, we identify a strong exotic QTL, associated with a favorable effect on yield under high salinity. The favorable effect of this QTL is due to the wild allele carried by lines in HEB family 1. Altogether, we describe the power of a barley NAM population developed using wild relatives of barley to locate yield-related salinity tolerance loci. We demonstrate that HEB-25 may be a valuable resource to develop improved elite barley cultivars that are better adapted to yield well under high salinity.

## Results and Discussion

The wild barley NAM population HEB-25 (Supplementary Table 1) was evaluated for ten traits related to agronomic performance and yield at ICBA (Supplementary Table 2). Plants were irrigated with low saline water, referred to hereafter as the control condition, or with saline water, referred to hereafter as the saline condition (Supplementary Figure 1). In addition to the ten traits measured in the field, we also derived indices for salinity tolerance (Supplementary Tables 3 and 4). The definition of what is desirable in regard to salinity tolerance in the field is not trivial. While lines that are salt tolerant *per se* are desirable, lines that are simultaneously capable of high yields under non-stress conditions are of practical agricultural importance. To differentiate lines in terms of stress tolerance and performance, stress-tolerance indices have previously been proposed^5^,^6^. For example, S/C is a simple index that indicates the tolerance of the plant to the stress regardless of the plant’s performance under non-stress conditions^5^. Another stress-tolerance index, STI, proposed by Fernandez^6^, enables identification of lines that are both stress tolerant and yield highly.

**Figure 1:**
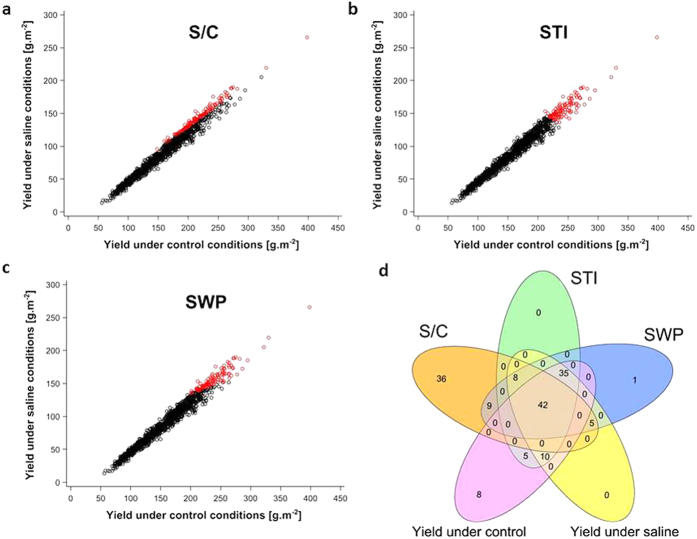
Comparison of the performance of three stress-tolerance indices, S/C, STI and SWP, using yield under saline conditions as a function of yield under control conditions. Red circles indicate the best performing 100 HEB lines identified by each stress-tolerance index ((**a**) S/C, (**b**) STI, (**c**) SWP) when lines are sorted by descending order for the index. Black dots indicate the remaining lines. (**d**) Venn diagram of shared/unique numbers of lines (out of the 100 top lines for each stress-tolerance index when lines are sorted in descending order) among the three indices and yield under saline and control conditions: numbers in the outer part of each oval correspond to the unique number of lines for each index while numbers in overlapping areas correspond to the number of shared lines that are identified by each index.

We propose a new index, stress-weighted performance (SWP, Supplementary Tables 3 and 4), which is capable of differentiating the top 100 high-yielding and salt-tolerant plants from other lines (Fig. 1). In SWP, lines with higher values are better performers under control conditions and are more salt tolerant than are lines with smaller values. SWP selects for lines that have above-average S/C and also have high yield under control conditions. This can be visualized in Fig. 1c, where lines with the top 100 values for this index have been colored red. SWP outperforms STI in stress-tolerance research because some lines that do not perform well but have above average S/C scores are identified by STI (Fig. 1b). Although STI captures the best lines based on yield performance (high yield), SWP captures the more salt-tolerant lines (Fig. 1c). In addition to the 42 lines common to all three indices seen in Fig. 1d, the salinity-tolerance index, S/C, has other lines in common with SWP, but not with STI. To achieve an improved identification of lines that are capable of high yield and also more tolerant to saline conditions, we thus advocate the use of SWP.

Salinity significantly reduces all traits measured in our field trials relative to control conditions (ANOVA, p < 2e-16 for all traits, Table 1). On average, yield is reduced by 43% under saline compared to control conditions.

**Figure 2:**
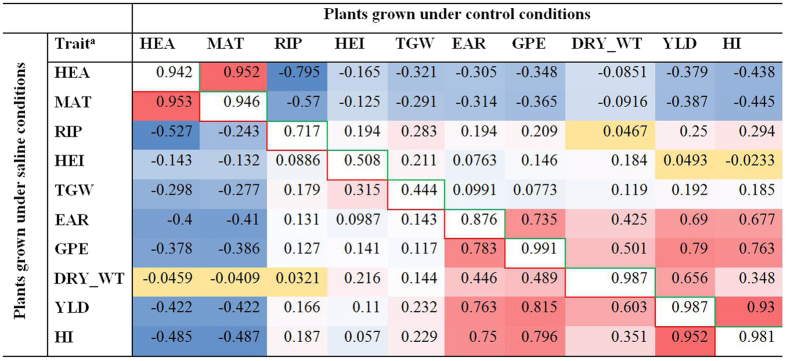
Pearson correlations between the ten studied traits. Correlations under control conditions are above the diagonal and correlations under saline conditions are below the diagonal. A heat map is used to color these correlations: blue indicates negative correlations, red indicates positive correlations, and the color intensity indicates the strength of the correlation (the darker the color the stronger the correlation). Within trait correlations between control and saline conditions are placed on the diagonal axis (white cells). All correlations are significant (p < 0.05) except for those in the yellow cells.^a^Flowering time (HEA), maturity time (MAT), ripening period (RIP), plant height (HEI), thousand grain mass (TGW), ear number per plant (EAR), grain number per ear (GPE), dry mass per m^2^ (DRY_WT), yield (YLD), and harvest index (HI).

**Figure 3:**
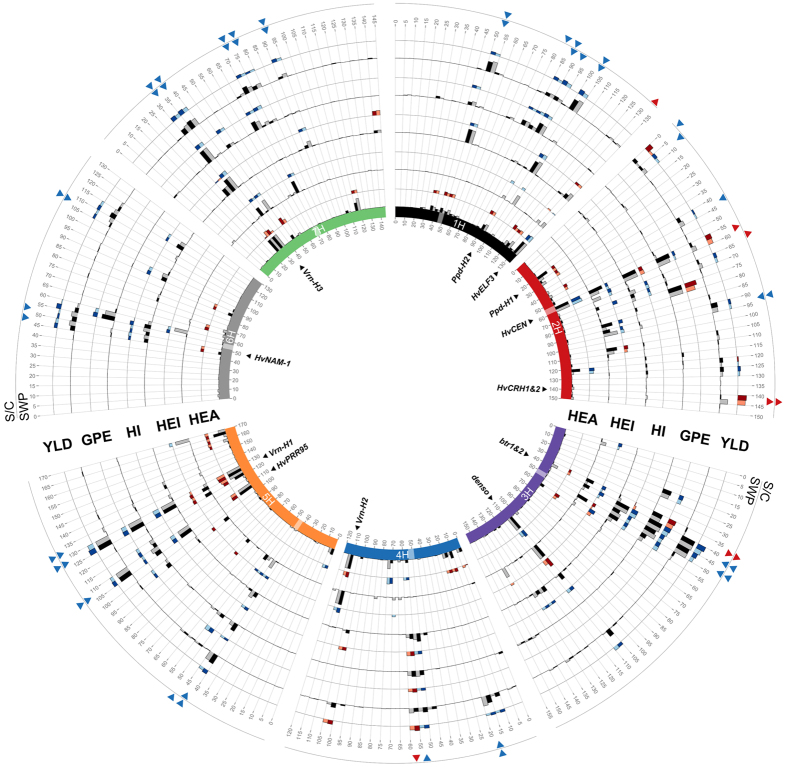
Genetic architecture of five of the studied traits: flowering time (HEA), plant height (HEI), harvest index (HI), grain number per ear (GPE), and yield (YLD). The data in this Circos plot results from 100 cross-validated (20 times 5-fold) GWAS runs performed within each treatment for the studied traits. Barley chromosomes are shown on the inner circle with different colors and centromeres are indicated with transparent boxes. For each trait, the first (inner) track represents the frequency of QTL detection in a 5-cM window while the outer track represents the effect of this QTL. The maximum height of the effect bars for each trait are 9 days for HEA, 17 cm for HEI, 0.1 for HI, 5 grains for GPE and 60 g.m^−2^ for YLD. Window positions (in cM, following Maurer *et al*.^4^) are ordered clockwise per chromosome. In the inner track, QTL appearing under control and saline conditions are represented with black and gray bars, respectively. The effect of the QTL conferred by the wild allele relative to Barke is represented on the outer track, where blue and red bars indicate decreasing and increasing wild barley QTL effects, respectively for each treatment. On the two outermost circles, significant QTL for stress tolerance indices (SWP and S/C, from inside to outside) are shown. Blue arrows pointing inwards and red arrows pointing outwards indicate decreasing and increasing effects from the wild barley alleles, respectively. Candidate genes, potentially explaining the observed QTL effects, are indicated inside the inner circle.

There is a strong correlation (|r| > 0.8) between the control and saline conditions in seven of the ten traits (flowering time, maturity time, ear number per plant, grain number per ear, dry mass per m^2^, yield and harvest index). Only ripening period, plant height, and thousand grain mass do not exhibit this correlation (Fig. 2 Supplementary Notes). This is reflected in the results presented in Fig. 3 where few quantitative trait loci (QTL) are shown to be treatment specific and most are significant under both control and saline conditions.

We observed loci controlling the flowering time trait with differing effects under control versus saline conditions, suggesting that these loci influence salinity tolerance. The significance of this observation is that flowering time negatively correlates with yield, yield components, and yield-derived traits (Fig. 2, Supplementary Notes). Under our conditions, salinity accelerates flowering by an average of 3.3 days. Here, we identify the genetic basis for this earlier flowering, which is associated with higher salinity tolerance.

Loci on chromosomes 1H (at 130–135 cM; cM positioning throughout this study follows Maurer *et al*.^4^) and 2H (at 55–60 cM), where *HvELF3*^7^ and *HvCEN*^8^ are respectively located, are significantly associated with flowering time under both control and saline conditions. The wild allele at the *HvELF3* locus causes earlier flowering and maturity under both control and saline conditions and increases the harvest index. The effect of the wild allele on promoting flowering under saline conditions is larger than it is under control conditions. Also, the extension of the ripening period caused by the wild allele is less pronounced under saline stress, allowing plants to complete their life cycles faster. We observe that the wild allele at the *HvELF3* locus decreases plant height and increases thousand grain mass under saline conditions only, which results in a significant increase in S/C, the salinity-tolerance index.

Similarly, the wild allele at the *HvCEN* locus promotes earlier flowering (by approx. 6 days) and earlier maturity (by approx. 4 days) under both control and saline conditions. It also reduces plant height and dry mass per m^2^. This indicates that the reduced resources invested in vegetative tissue may enable the increased number of ears and increased yield (by 21 g.m^−2^). The reduction in grain number per ear could be related to the production of bigger grains per ear or more tillers per plant. The effect of the wild allele on reducing dry mass per m^2^ and increasing yield results in an overall increasing effect on the harvest index. We observe that the salinity tolerance index, SWP, but not S/C, is significantly increased at the *HvCEN* locus, indicating that this locus is associated more with better yield performance than with increased salinity tolerance. We conclude that *HvCEN* may be of great interest to breeders because of its yield improvement under both control and saline conditions.

In addition to loci that improve yield by accelerating flowering, we found a strong QTL on chromosome 2H, 140–145 cM with a direct favorable effect on yield (increasing by 33 g.m^−2^, 21% under control conditions and 37% under saline conditions) that is conferred by the wild barley allele. This QTL appears in cross-validations 56 times under salinity and eight times under control conditions. The SNP showing the significant yield effect is BOPA2_12_30822 in the *alpha-glucosidase* gene (AK375658). This SNP segregates only in HEB family 1, explaining 19.8% of the phenotypic variation in yield under high salinity in this family. In HEB family 1, lines carrying the wild barley allele at this SNP have a significantly higher yield under both control and saline conditions than do lines carrying the Barke allele at this SNP (Table 2). Both S/C and SWP indices are significant, revealing a favorable effect of the wild allele at this locus. Interestingly, this QTL is only significant for yield, which implies that each yield component only slightly increases. Moreover, because both the S/C and SWP indices for yield at this QTL are also significant, we suggest that this QTL increases the salinity-tolerance component of SWP. Hence, the wild barley donor of HEB family 1, HID_003, seems to confer an allele that increases yield under high salinity. However, the A2148G substitution that is detected by the SNP does not affect the amino acid sequence. A second SNP, SCRI_RS_116590, is located at the same position as BOPA2_12_30822 (2H, 140.8 cM) and segregates in 24 out of 25 HEB families. However, the yield effect that we observe between lines homozygous for wild and Barke alleles at BOPA2_12_30822 is not observed at SCRI_RS_116590 (Table 2). This result indicates that the favorable yield effect observed with BOPA2_12_30822 may be restricted to donor HID_003, originating from northeastern Iraq.

Genes related to the barley *alpha-glucosidase* gene in this QTL have been studied in other species. It has been shown that the Arabidopsis *alpha-glucosidase* gene is involved in cell wall biosynthesis^9^ and in the first steps of N-glycan trimming in the endoplasmic reticulum^10^. Furthermore, a study of knockout mutants of Arabidopsis *alpha-glucosidase* suggested that the N-glycosylation of this gene was required for salinity tolerance^11^. Further experimental evidence, involving manipulation of *alpha-glucosidase* expression, is required to test whether this gene is the main cause of the favorable yield effect observed in our study. Alternatively, this gene could be linked to the causative gene responsible for the phenotype observed in family 1. Interestingly, barley calreticulin 1 (*CRH1*, GenBank: L27348.1) and calreticulin 2 (*CRH2*, GenBank: L27349.1), which are calcium-binding proteins, align to position 141.78 cM and may instead be the genes underlying this QTL (2H, 140–145 cM). Xiang *et al*.^12^ have shown that tobacco plants transformed with *Triticum aestivum* calreticulin gene (*TaCRT1*) showed higher salinity tolerance compared to their wild-type counterparts. Another candidate in the region of interest is a choline-transporter-like gene (MLOC_63287.1). Enzymes mediating choline oxidoreduction have also been shown to be involved in salinity tolerance^2^. Choline is converted by choline dehydrogenase/oxidase to glycine betaine^13^,^14^, an osmoprotectant known to increase salinity tolerance^2^.

In several studies, analyses of transcriptomes of wild and cultivated barley grown under control and saline conditions have been conducted to identify differential gene expression in response to salinity^15^,^16^,^17^,^18^,^19^,^20^. Several members of ubiquitous gene families that were reported to be differentially expressed in those transcriptomic studies, could be located within the QTL region on chromosome 2H, 140–145 cM. These genes code for: pentatricopeptide repeat domain-containing proteins, RNA recognition motif containing proteins, zinc finger proteins, MYB family transcription factors, heat shock hsp70 proteins and cytochrome P450. However, no further evidence is available that would support a direct involvement in the salinity tolerance of HEB family 1. Also, it needs to be remembered that genes whose expression changes in response to salinity stress may not necessarily contribute to salinity tolerance.

In addition, Wu *et al*.^21^ compared the ionic and proteomic responses of a wild barley genotype, XZ16, and a more salt-tolerant cultivar, CM72, to salinity under hydroponic conditions. The authors found that putative class III peroxidases (GenBank: AK365672 and AK363236) were significantly upregulated in the roots of both genotypes. Based on barley genome sequence blasting, we could map these genes to chromosome 2H, 146.5cM. Given the known role of reactive oxygen species (ROS) and the ROS-scavenging system in salinity tolerance, it is conceivable that the putative class III peroxidase genes are candidates that could explain the function of the QTL on chromosome 2H, 140–145 cM. Other loci controlling yield are identified in the Supplementary notes (included in the Supplementary Information).

Using our new stress-tolerance index, SWP, on HEB-25, we identified genes putatively affecting flowering time and salinity tolerance in barley. We also located a QTL responsible for higher yield. Further studies are necessary to validate the candidate genes and to study in more depth the mechanisms leading to increased yield and salinity tolerance with the ultimate goal of introgressing salinity tolerance traits (loci) into commercial barley lines. Studying the causal genes in other crops, notably wheat, also provides significant opportunities to improve performance of major crops in saline conditions, guided by this work in the relatively salt-tolerant crop, barley.

## Materials and Methods

### Plant material

HEB-25, a barley NAM population, comprises 25 families resulting from crosses of the German spring cultivar Barke with 25 wild donors, 24 *H. vulgare* ssp. *spontaneum* (*Hsp*) from the Fertile Crescent and one Tibetan *H. vulgare* ssp. *agriocrithon* (*Hag*) accession. The resulting F_1_ plants were backcrossed with Barke as a female parent and then selfed three times through the application of a single seed descent procedure. Subsequently, BC_1_S_3_ lines were bulk propagated to BC_1_S_3:6_. Further details on HEB-25 are provided in Maurer *et al*.^4^. In this study, 1,336 BC_1_S_3:7_ lines of HEB-25 (Supplementary Table 1) were tested in the field under control and saline conditions.

### Field trials

A field trial was conducted for two years at the International Center for Biosaline Agriculture (ICBA), Dubai, United Arab Emirates (N 25° 05.847; E 055° 23.464), from December to May (2013–2014 and 2014–2015).

The soil at ICBA is a fine sand (sand 98%, silt 1%, and clay 1%), calcareous (50–60% CaCO_3_ equivalents), porous (45% porosity), and moderately alkaline (pH 8.22). The saturation percentage of the soil is 26; it has a high drainage capacity; and the electrical conductivity of its saturated extract (ECe) is 1.2 dS.m^−1^. ICBA soil is classified as Typic Torripsamments, carbonatic, and hyperthermic^22^,^23^. Koblenz Organic Fertilizer (manufactured by Tadweer Waste Treatments LLC (Dubai, UAE) was added to the top of the soil at a rate of 40 tonnes FW.ha^−1^ (at 85% moisture) to increase the soil water-holding capacity and to provide some of the required nitrogen (N), potassium (K), sulfur (S), and micro-nutrients. Two weeks before sowing, fertilization with phosphorus (P) was conducted using a single supply of 45 kg P_2_O_5_ ha^−1^ (100 kg of Triple Super Phosphate from Benedict (Indonesia) with a total of 45% phosphoric anhydride). Granular urea nitrogen (N) fertilizer from Fertil (Abu Dhabi, UAE) was applied once at a rate of 30 kg N ha^−1^ (i.e. 3 g.m^−2^ pure N, equivalent to 7.5 g urea per plot at 46% N), three weeks after planting. An application of NPK fertilizer (20-20-20) from ADFERT (Abu Dhabi, UAE) at a rate of 30 kg.ha^−1^ was also made six weeks after planting by fertigation.

The 1,336 NAM lines were grown, along with Barke and the 25 wild donors, irrigated with saline (referred to as the saline condition throughout the paper) and low-saline water (referred to as the control condition throughout the paper). Plots were randomized in an augmented design, and salt-tolerant check lines (116/2A, 58/1A, CM72) were sown every seven plots on average. Four rows of a local barley cultivar (58/1A) were sown around the experimental area to reduce edge effects. The plot size was 1.5 m × 0.5 m, containing five rows, each 25 cm apart. Approximately 25 seeds (at 2 cm spacing and 1 cm depth) were hand-sown per row (Supplementary Figure 1).

The plots were irrigated twice per day for ten minutes each time. Each plot received 13.3 L water per day. Control plots were irrigated with water of 1 dS.m^−1^; and saline plots were irrigated with 1 dS.m^−1^ water during the first week followed by irrigation with saline water (17 dS.m^−1^) for the remainder of the growing period. The ionic composition of the irrigation water was monitored throughout the season using salinity sensors. The distribution of drippers was homogeneous and the distance between drippers allowed the overlapping of wetting fronts. Weather data was recorded at the field site at ICBA during the two years of field trials (Supplementary Table 5).

Ten agronomic traits were measured under control and saline conditions (Supplementary Table 2) and stress-tolerance indices were derived from those traits (Supplementary Table 3).

### Genotyping of HEB-25

Genotyping of HEB-25, along with the parents, was performed using an Illumina Infinium iSelect HD 9k chip consisting of 7,864 SNPs as previously described^8^. The 5,398 informative SNPs that were polymorphic in at least one HEB family and that met certain quality criteria (<10% missing, <12.5% heterozygous, and not in complete linkage disequilibrium (LD) to another SNP in the set) were kept for further analyses. Further details on the genotypic data are available in Maurer *et al*.^4^.

### Statistical analysis of phenotypic data

To correct for spatial variation in the field, a multi-environment trial (MET) analysis was conducted for two years on each trait and predicted line means adjusted for environmental variation were obtained (Supplementary Dataset 1). In the MET analysis, each year by treatment combination was considered as a separate environment or trial, with these trials correlated in a MET analysis following the factor analytic model of.Smith *et al*.^24^. The environmental variation often present in field trials was modeled according to the work of Gilmour *et al*.^25^ that allowed for the three possible sources of environmental variation: global, extraneous, and local. The need for adjustment for environmental variation was explored by examination of residual plots including a variogram. The environmental terms identified were then added to the MET analysis model.

Consider a data set consisting of *v* lines and *s* trials, the latter of which correspond to the two-treatment-by-two-year combinations with ***y*** the vector of response, an appropriate linear mixed model for a MET analysis is as follows:


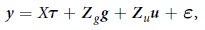


where 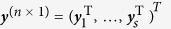 and 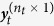 is the vector of response for trial *t* and 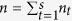, where *n*_*t*_ is the number of observations (plots) in trial *t*, ***τ*** is a vector of fixed terms consisting of an overall mean performance for each trial as well as trial specific global or extraneous trial terms such as linear row or linear column trends, ***X*** is the associated design matrix, 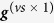 is the vector of random line effects of the *v* lines in each of the *s* trials with design matrix ***Z***_*g*_ and associated variance var(***g***)
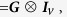
where ***I***_*v*_ is the (*v* × *v*) identity matrix, 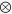 is the Kronecker product, ***G*** is the (*s* × *s*) genetic variance matrix. Here we consider a factor analytic structure for ***G***^24^, with two factors, ***G*** = ***ΛΛ***^***T***^ + ***Ψ***, with **Λ** being a matrix of factor loadings at each of the *s* trials, and ***Ψ*** a diagonal matrix with elements relating to specific variances for trial s. This formulation for ***G*** allows a separate variance for each trial and a difference covariance between pairs of trials. ***u*** is a vector of random effects and includes extraneous environmental variation specific to each trial such as random row or column effects or a random smoothing spline^26^ and ***Z***_*u*_ is the associated design matrix. The residual vector, 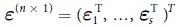, represents local stationary variation at the *t*th trial with 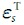 having variance 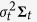 for 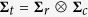, where **Σ**_*t*_ represents the Kronecker product between auto-regressive processes of order one (AR1) in the column and row directions for the *t*th trial.

The generalized heritability was calculated for each trial separately (i.e. assuming ***G*** = ***Ψ***) as 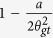 where *a* is the average pairwise prediction error variance of line effects and 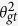 is the specific genetic variance of trial *t*^27^. Results are shown in Supplementary Table 6. The factor analytic models were fitted in ASReml v3.0-1^28^ for R v3.2.0^29^.

A linear mixed model was fitted to calculate best linear unbiased estimates (BLUEs) across years of the adjusted values per plot to take into account the year and line-by-year interactions. The adjusted HEB means across years were used in the GWAS.

Traits and indices were correlated using Pearson correlation, and analysis of variance (ANOVA) was used to study the effect of treatment on each trait.

### Association mapping analysis

To conduct GWAS, we used the multiple linear regression Model-B of Liu *et al*.^30^, in which cofactors and a population effect were included in addition to the SNP under investigation. This model exhibited high predictive power in previous studies and effectively controlled for population structure when compared to other joint linkage association mapping models^31^. This model has been shown to perform well in the Maurer *et al*.^4^ study of flowering time in the HEB-25 population.

The analysis was conducted with SAS 9.4 Software (SAS Institute Inc., Cary, NC, USA) using *Proc GLMSELECT*. Significant SNPs were determined by stepwise forward-backward regression. SNPs were allowed to enter or leave the model at each step if p < 0.001. To estimate the proportion of phenotypic variance explained by each significant SNP, R^2^ was calculated after modelling the SNP solely in a linear model. Additive effects of the wild allele relative to the Barke allele were estimated across families and were taken as the regression coefficient of the SNP from the GWAS model. Multiplication of those additive effects by a factor of two represents the absolute difference between the wild allele effect and the Barke allele, which is referred to throughout the paper.

A five-fold cross-validation was run 20 times to increase the robustness of the GWAS. The 100 subsets were taken from the total phenotypic dataset. Each subset consisted of 80% randomly chosen HEB lines used as a training set to define significant SNPs and the remaining 20% of lines were used as a test set. The phenotype of these latter lines was predicted based on the SNP effects estimated in the training set. ‘R^2^val’ was then calculated as the squared Pearson product, the moment correlation between predicted and observed phenotypes in the test set, whereas ‘R^2^train’ represents the model fit of the training set. The detection rate was calculated as the number of times, out of 100, that a SNP showed significance (results in Supplementary Table 7 and Supplementary Dataset 2). To enable comparison of shared QTL regions across cross-validation runs, detection rates were accumulated and SNP effects were averaged within 5 cM windows, as shown in the Circos plot of Fig. 3. Individual Manhattan plots for each trait and condition were created with qqman^32^ (Supplementary Figure 2). The −log_10_(p-values) were averaged across all 100 cross-validation runs and the mean was weighed through multiplying by the sum of occurrences (out of 100) and dividing the overall result by 100. The resulting numbers were referred to as cross-validated −log10(p) in Supplementary Figure 2. The Bonferroni-adjusted 5% significance threshold was calculated according to Holm^33^. This threshold has been weighed by a factor of 0.2, reflecting a detection rate of 20 out of 100 cross-validation runs.

## Additional Information

**How to cite this article**: Saade, S. *et al*. Yield-related salinity tolerance traits identified in a nested association mapping (NAM) population of wild barley. *Sci. Rep.* **6**, 32586; doi: 10.1038/srep32586 (2016).

## Supplementary Material

Supplementary Information

Supplementary Dataset 1

Supplementary Dataset 2

## Figures and Tables

**Table 1:** Agronomic performance of HEB-25 lines and Barke under control and saline conditions.

Trait^a^	Condition	Barke	HEB-25 population		
		Mean	Mean	SE^b^	CV (%)^c^
HEA (days)	Control	80.6	82.6	0.221	9.79
	Saline	77.2	79.3	0.220	10.1
MAT (days)	Control	110	112	0.163	5.35
	Saline	104	106	0.193	6.64
RIP (days)	Control	29.4	29.1	0.0827	10.4
	Saline	26.3	26.8	0.0690	9.40
HEI (cm)	Control	75.1	75.1	0.334	16.3
	Saline	66.4	63.1	0.202	11.7
TGW (g)	Control	40.3	37.8	0.140	13.5
	Saline	35.3	33.2	0.115	12.7
EAR	Control	4.84	3.94	0.0270	25.0
	Saline	3.77	2.50	0.0158	23.1
GPE	Control	20.0	14.5	0.0885	22.3
	Saline	15.9	10.6	0.0746	25.8
DRY_WT (g.m^−2^)	Control	627	511	1.59	11.4
	Saline	521	422	1.48	12.9
YLD (g.m^−2^)	Control	254	157	1.27	29.5
	Saline	165	89.3	0.978	40.0
HI	Control	0.404	0.305	0.00196	23.6
	Saline	0.320	0.210	0.00194	33.8

^a^Flowering time (HEA), maturity time (MAT), ripening period (RIP), plant height (HEI), thousand grain mass (TGW), ear number per plant (EAR), grain number per ear (GPE), dry mass per m^2^ (DRY_WT), yield (YLD), and harvest index (HI).

^b^SE stands for standard error.

^c^CV stands for coefficient of variation.

**Table 2:** Comparison of yield performance between lines by field condition (control and saline) and by genotype at SNPs BOPA2_12_30822 and SCRI_RS_116590.

Condition	Genotype at SNP^a^	BOPA2_12_30822^b^ at 2H, 140.8 cM		SCRI_RS_116590^c^ at 2H, 140.8 cM	
		Yield average (g.m^−2^) ± standard deviation	p value^d^	Yield average (g.m^−2^) ± standard deviation	p value^d^
Control	0	180 ± 39.7	0.00323	158 ± 45.8	0.163
	1	205 ± 19.3		150 ± 42.4	
	2	216 ± 43.3		153 ± 48.8	
Saline	0	107 ± 31.6	0.0009	89.6 ± 35.1	0.145
	1	128 ± 16.4		84.2 ± 33.6	
	2	139 ± 34.8		86.3 ± 38.1	

^a^Values 0 and 2 indicate HEB lines homozygous for Barke and wild barley alleles, respectively, and value 1 indicates heterozygous HEB lines.

^b^BOPA2_12_30822 only segregates in HEB family 1, with 29 vs 23 vs 3 lines carrying the genotypes homozygous Barke, homozygous wild barley and heterozygous, respectively.

^c^SCRI_RS_116590 segregates in 24 out of 25 HEB families (i.e. all except HEB family 24), with 868 vs 317 vs 91 lines carrying the genotypes homozygous Barke, homozygous wild barley, and heterozygous, respectively.

^d^p value of the t-test to check differences in yield average between lines carrying the genotype homozygous Barke and lines carrying the genotype homozygous wild barley.
