# Supplementary material for: Yield-related salinity tolerance traits identified in a nested association mapping (NAM) population of wild barley
Source: Sci Rep. 2016 Sep 2;6:32586. doi: 10.1038/srep32586 (PMC5009332; doi:10.1038/srep32586)
Supplement: Supplementary Information [file srep32586-s1.pdf]

Supplementary information

Yield-related salinity tolerance traits identified in a nested association mapping (NAM) population of wild barley

Stephanie Saade, Andreas Maurer, Mohammed Shahid, Helena Oakey, Sandra M. Schmöckel, Sónia Negrão, Klaus Pillen & Mark Tester

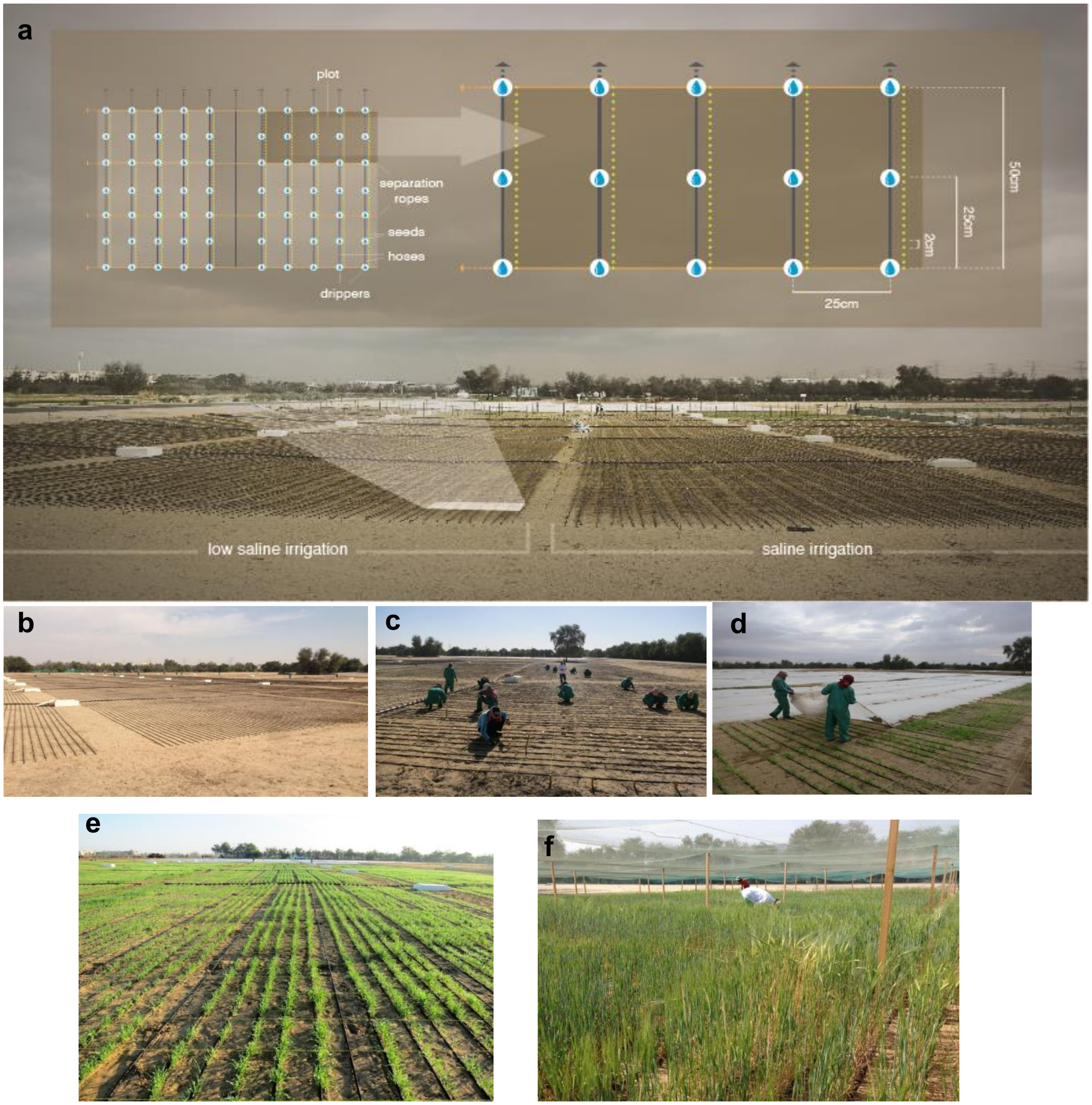

Supplementary Figure 1: Field experiment at the International Center for Biosaline Agriculture (ICBA) in Dubai, United Arab Emirates.

(**a**) Photograph of the experimental field site at ICBA, with irrigation and plot dimensions indicated in the image. A plot consists of five rows; only the three middle rows of each plot were used for physiological measurements, with an area of 0.5 * (0.25+0.25+0.25) = 0.375 m^2. (**b**)

Water pumps (white boxes) provide drip irrigation through hoses on the sandy soil. (**c**) Manual sowing along the hoses. The seeds are sown at around 1 cm depth. (**d**) The plots were covered with breathable, loosely woven polyester fabric sheeting during germination to protect the seedlings from birds. (**e**) Plants at the seedling stage. (**f**) Plants at the flowering stage.

Supplementary Figure 2: Manhattan plots for each trait under control and saline conditions. Cross-validated $–\log_{10}(p)$, represented by dots, were calculated by averaging $–\log_{10}$(p-values) across all runs then weighing the mean by multiplying with the sum of occurrences (out of 100) and dividing the overall result by 100. The red dot represents BOPA2_12_30822, the peak SNP of the QTL (2H, 140-145 cM) for yield under saline conditions. The chromosomal and positional assignment corresponds to the map of Maurer, et al. [1].The red line marks the weighed Bonferroni-adjusted 5% significance threshold[2].

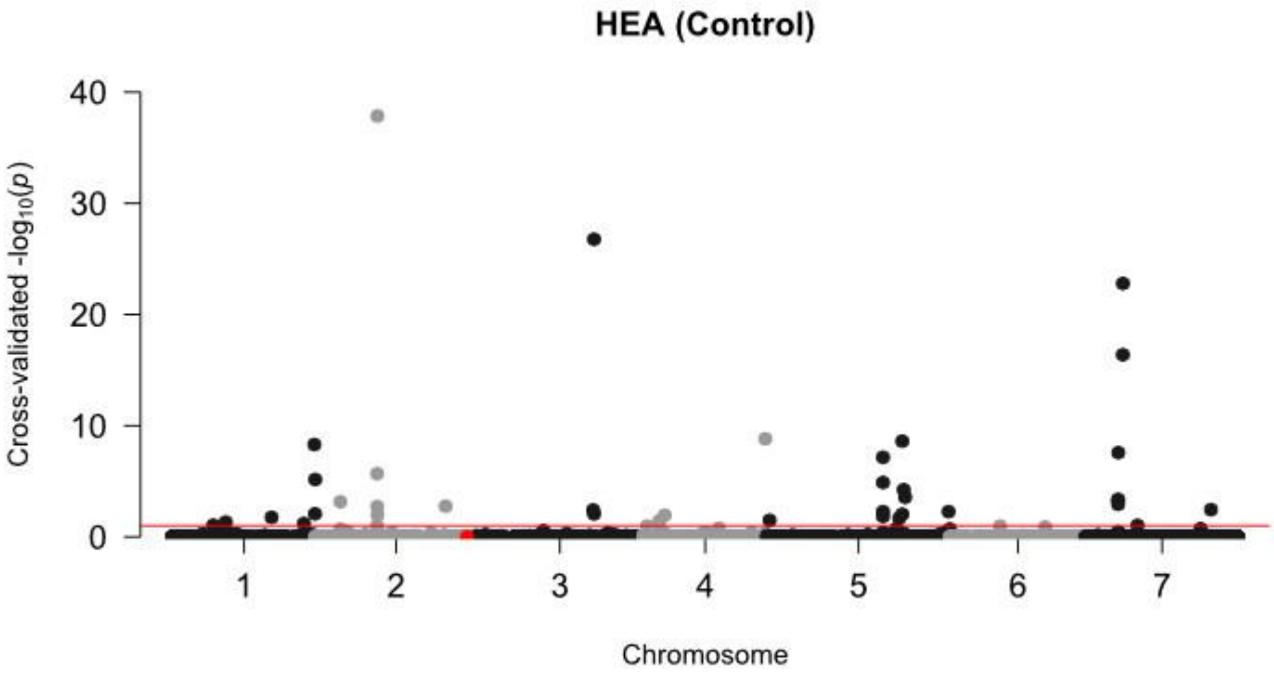

a) Manhattan plot for flowering time under control conditions

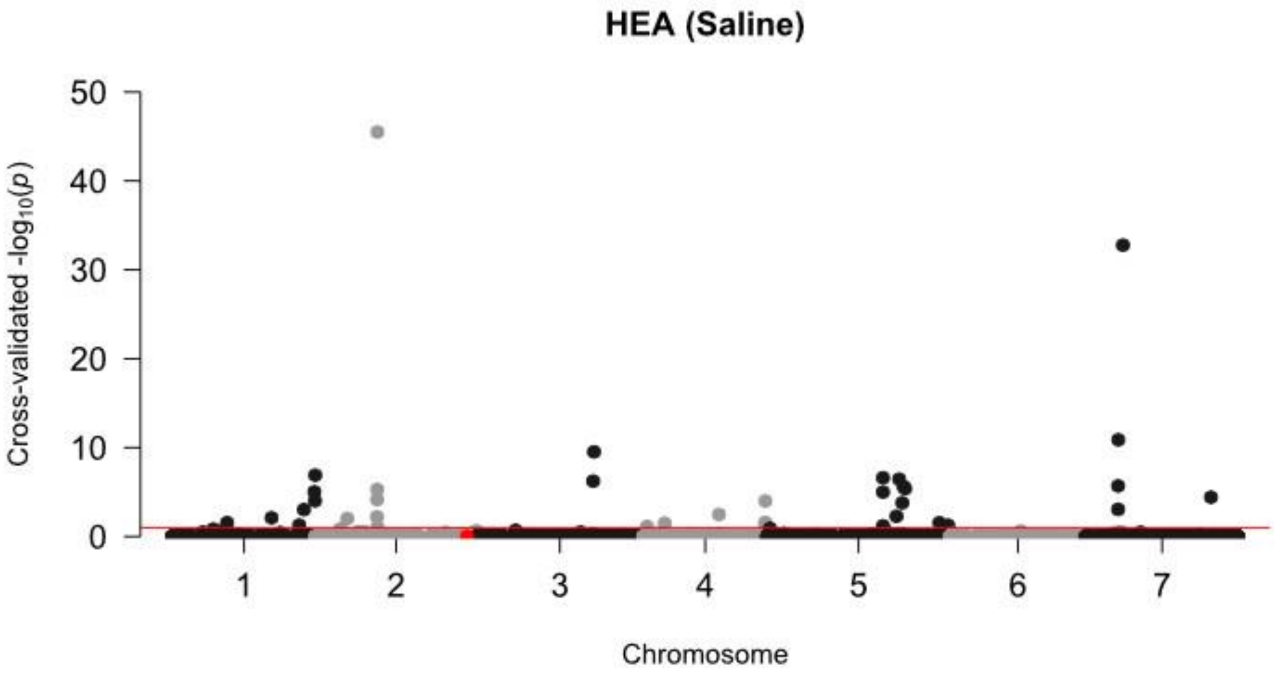

b) Manhattan plot for flowering time under saline conditions

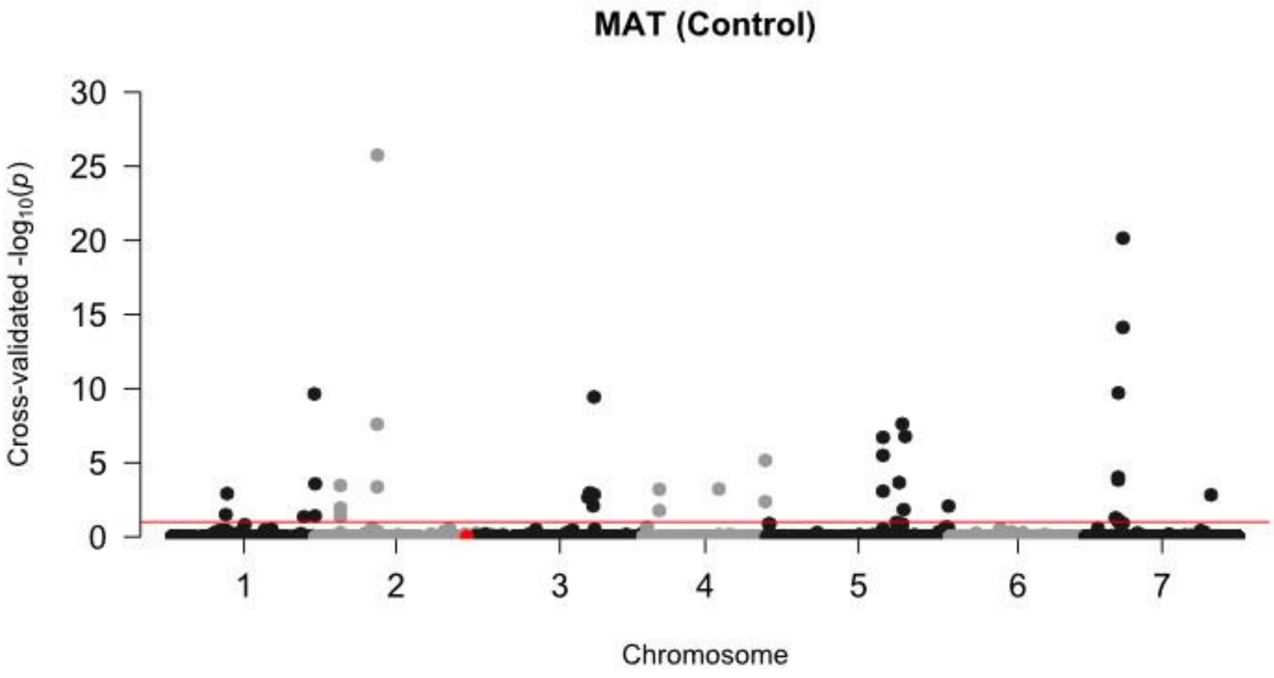

c) Manhattan plot for maturity time under control conditions

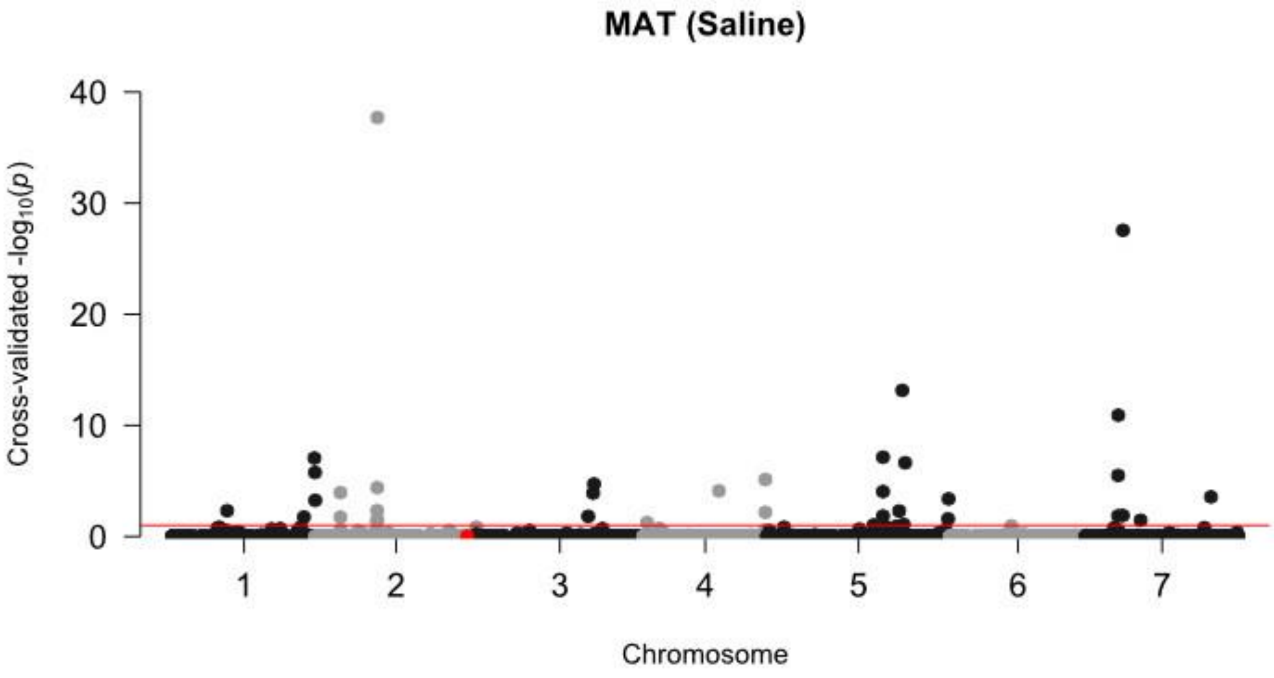

d) Manhattan plot for maturity time under saline conditions

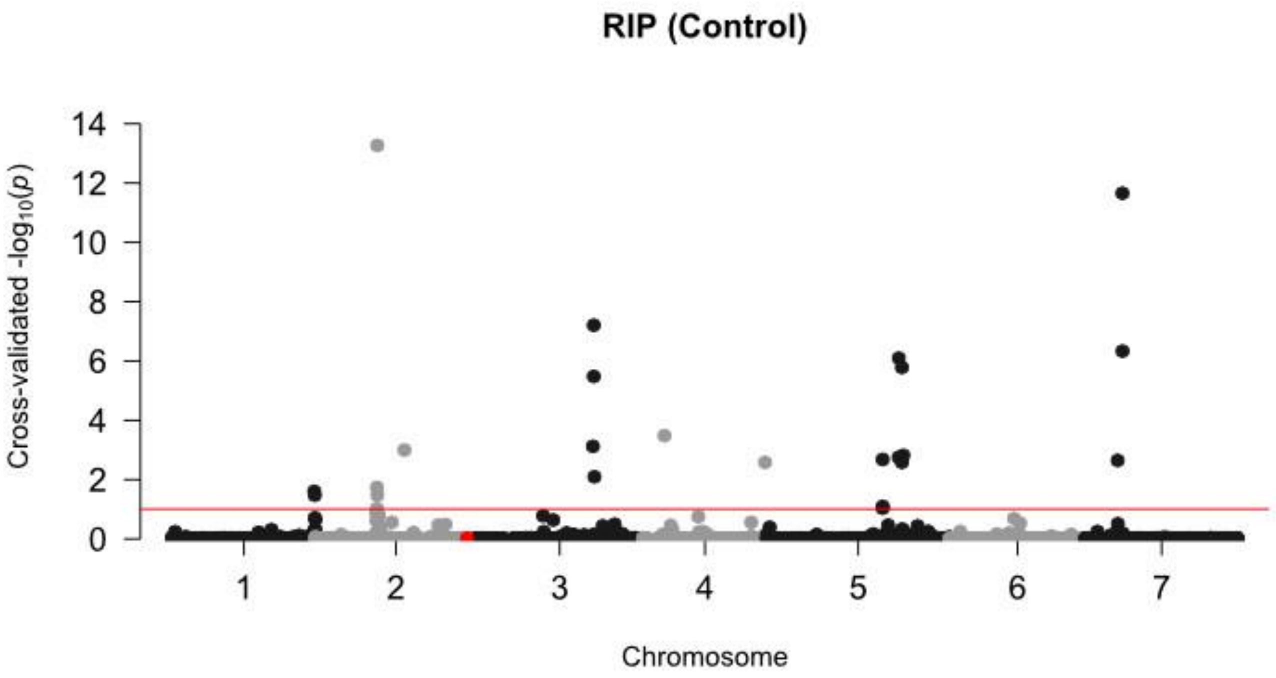

e) Manhattan plot for ripening time under control conditions

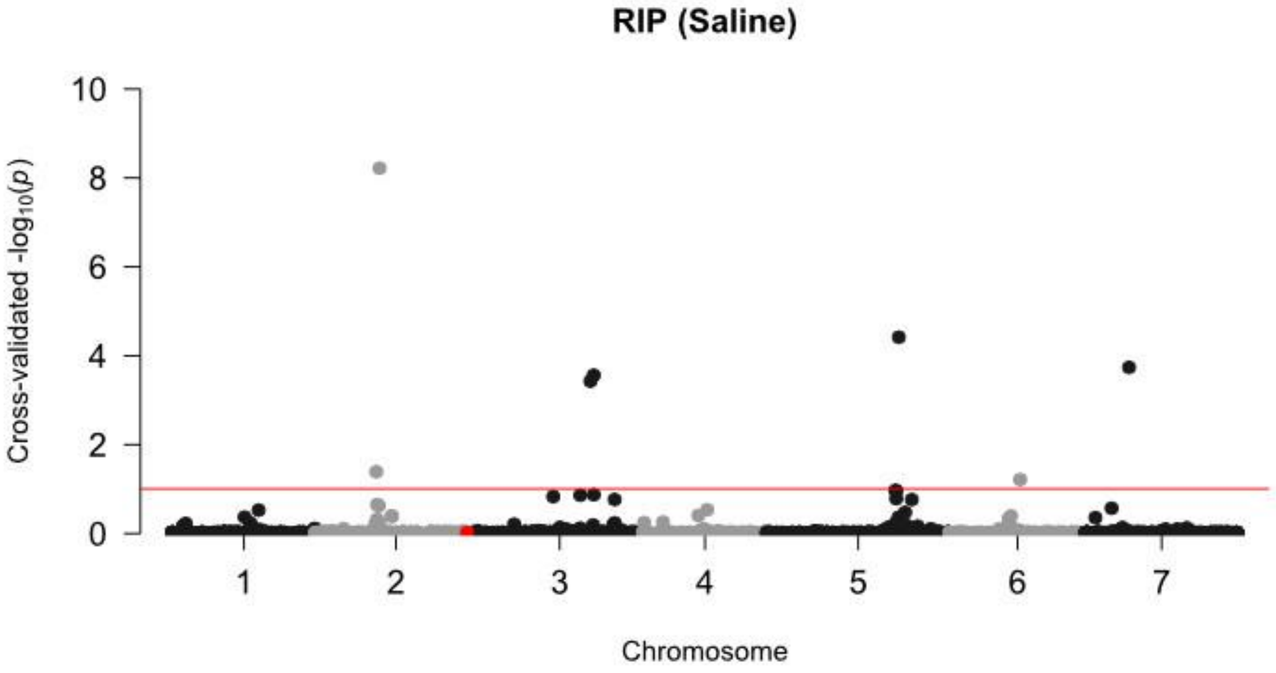

f) Manhattan plot for ripening time under saline conditions

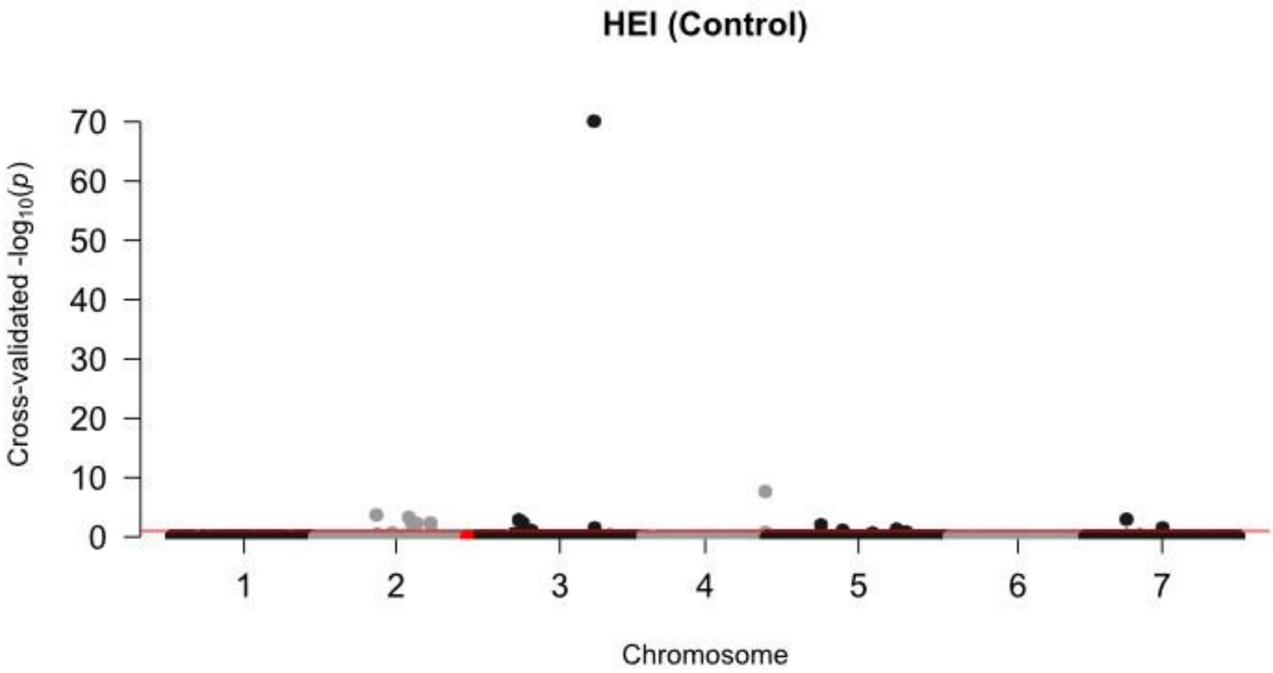

g) Manhattan plot for height under control conditions

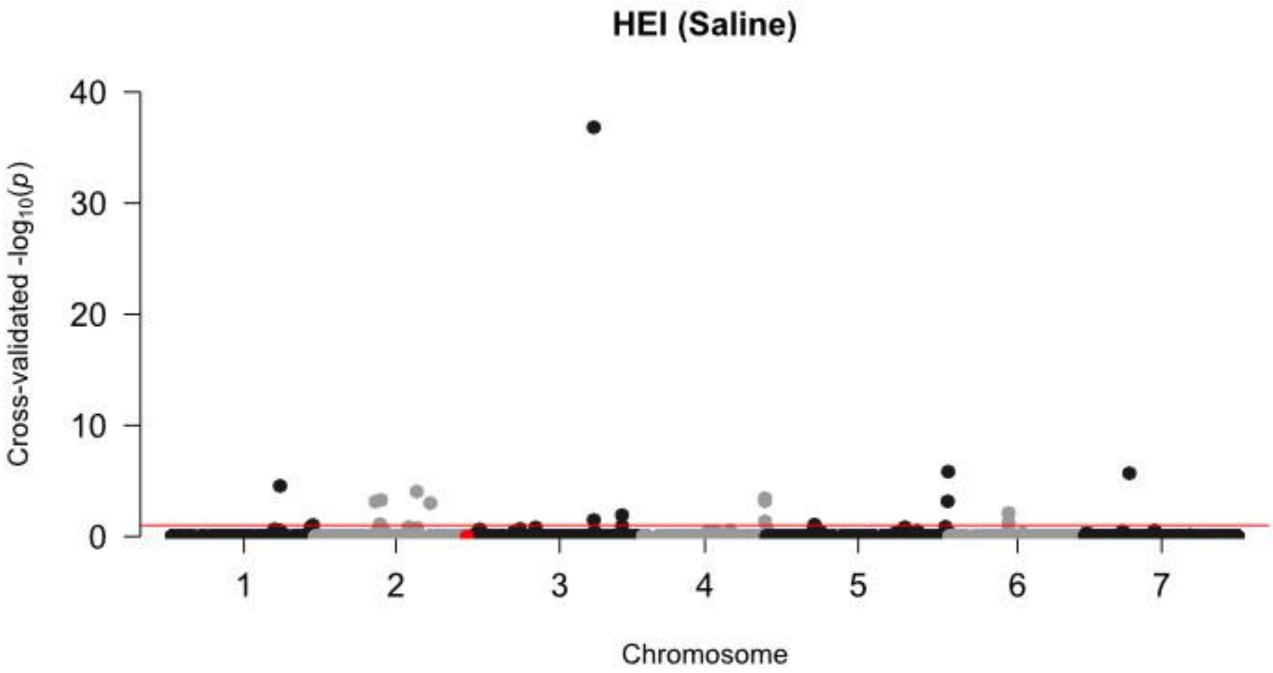

h) Manhattan plot for height under saline conditions

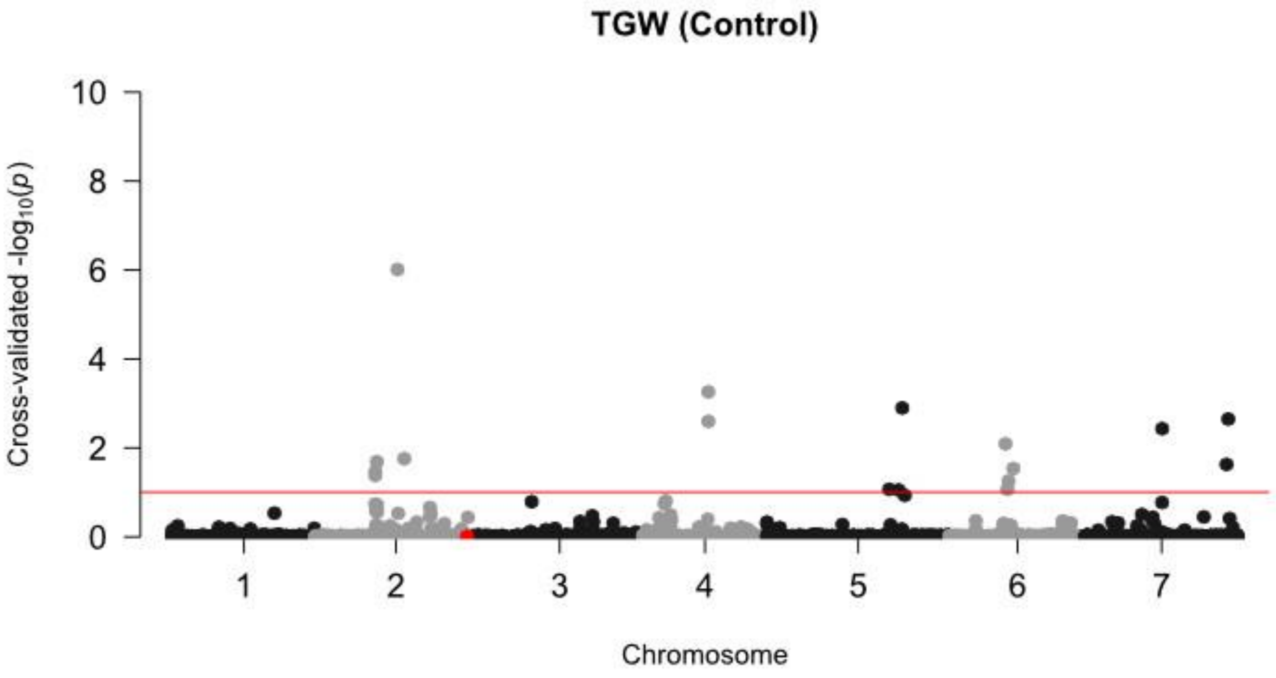

i) Manhattan plot for thousand grain mass under control conditions

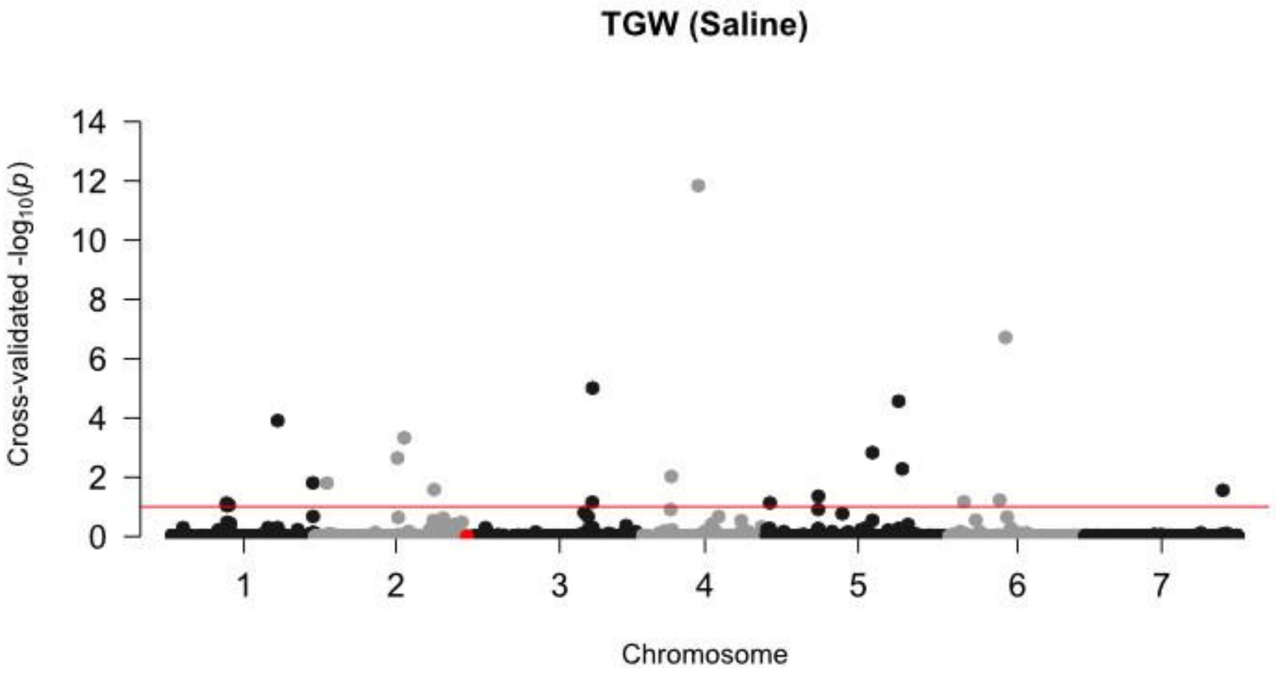

j) Manhattan plot for thousand grain mass under saline conditions

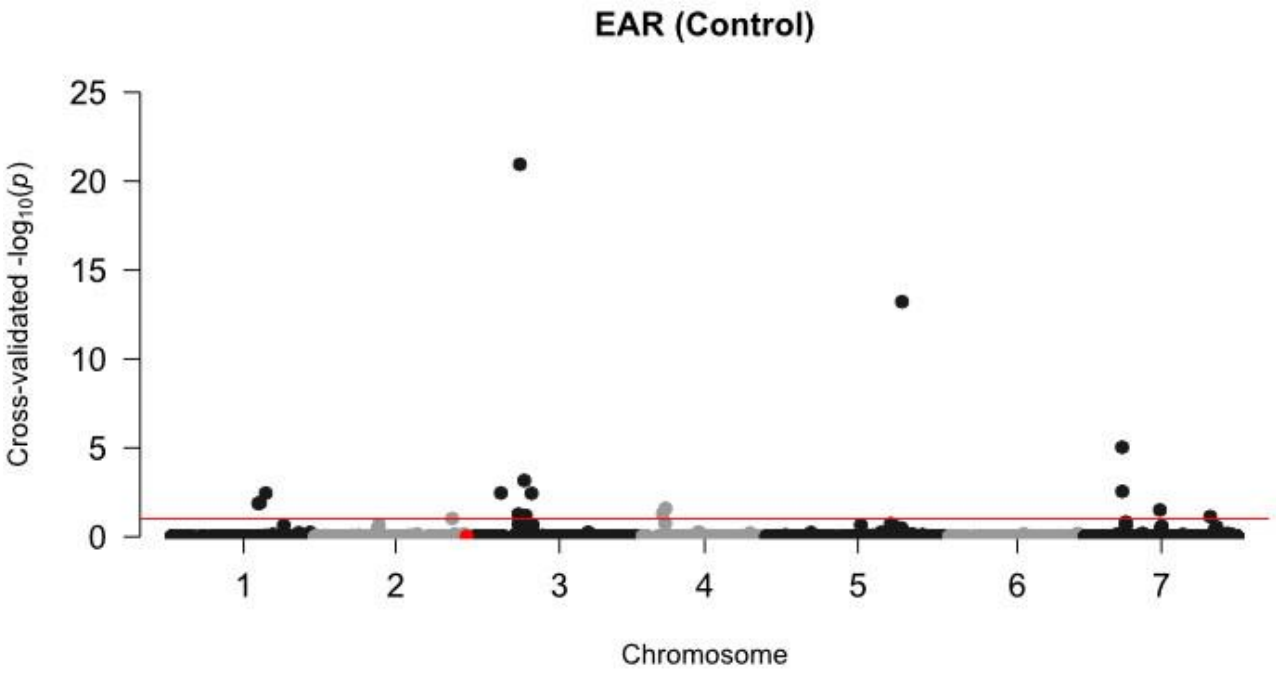

k) Manhattan plot for ear number per plant under control conditions

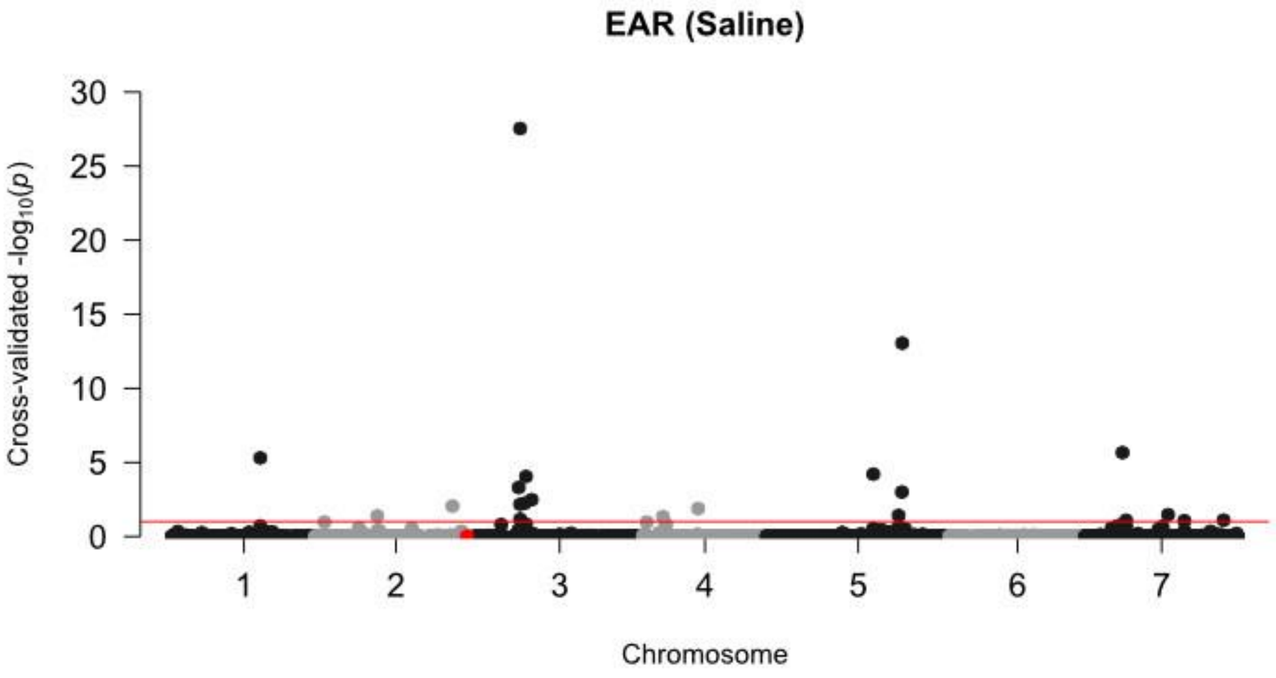

l) Manhattan plot for ear number per plant under saline conditions

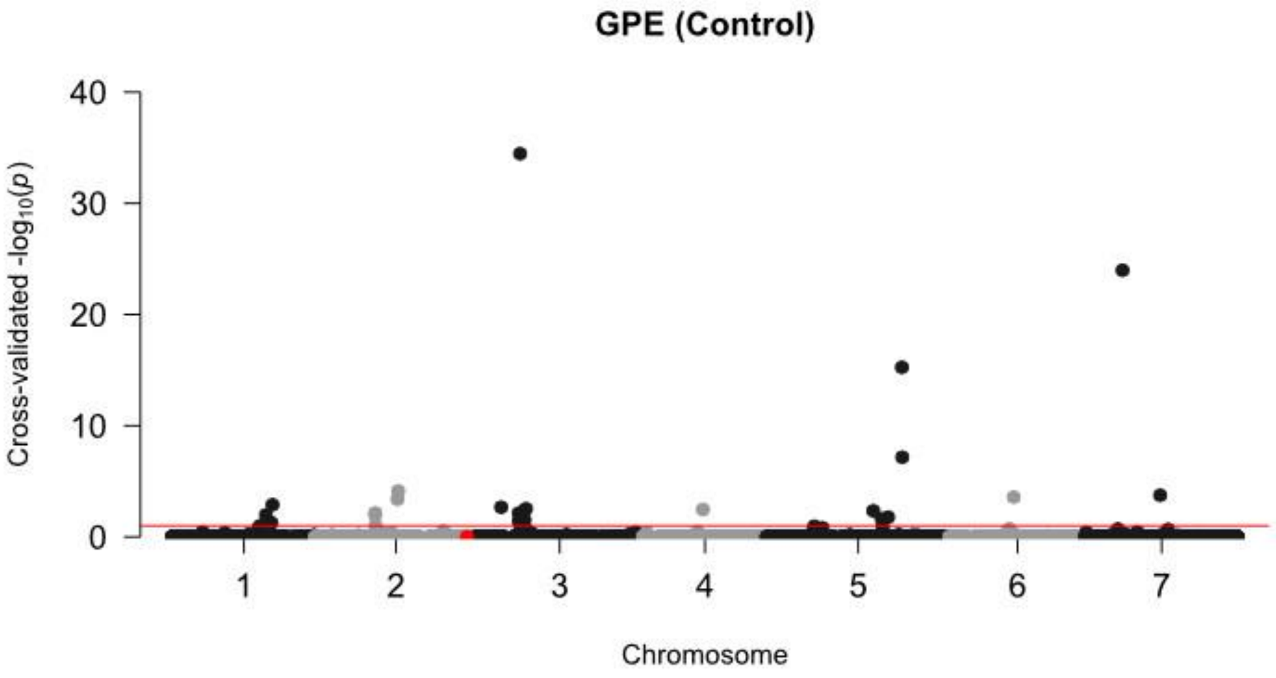

m) Manhattan plot for grain number per ear under control conditions

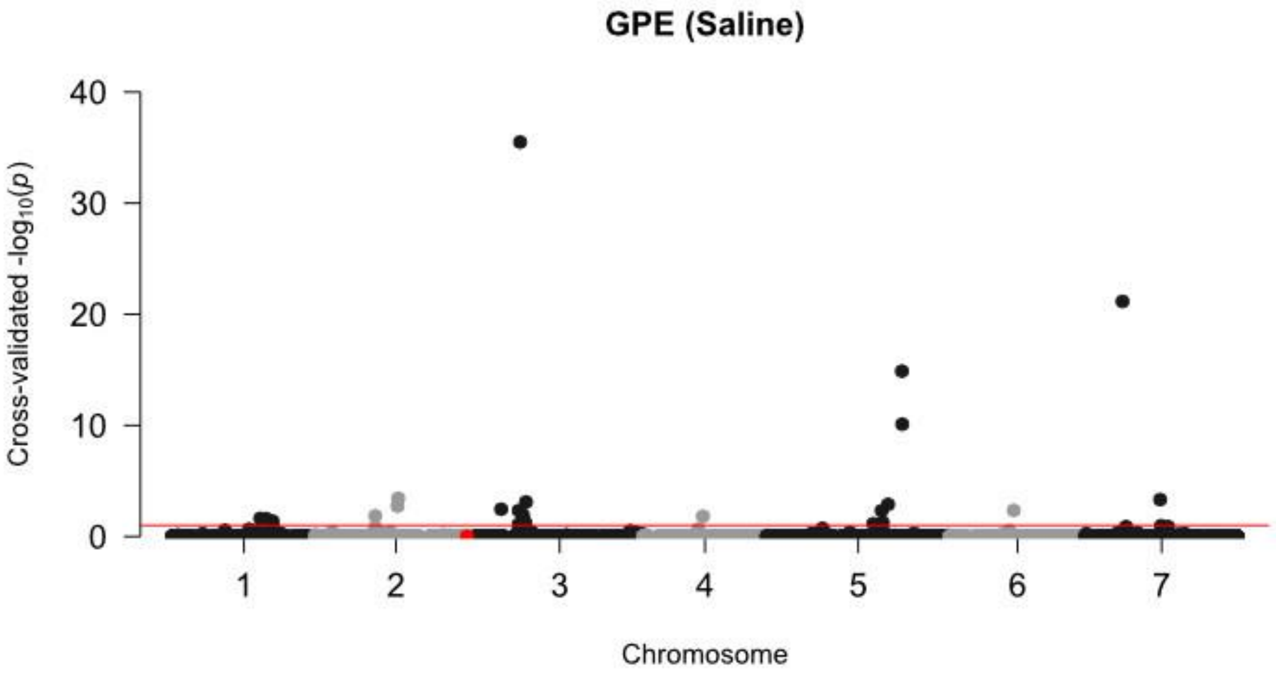

n) Manhattan plot for grain number per ear under saline conditions

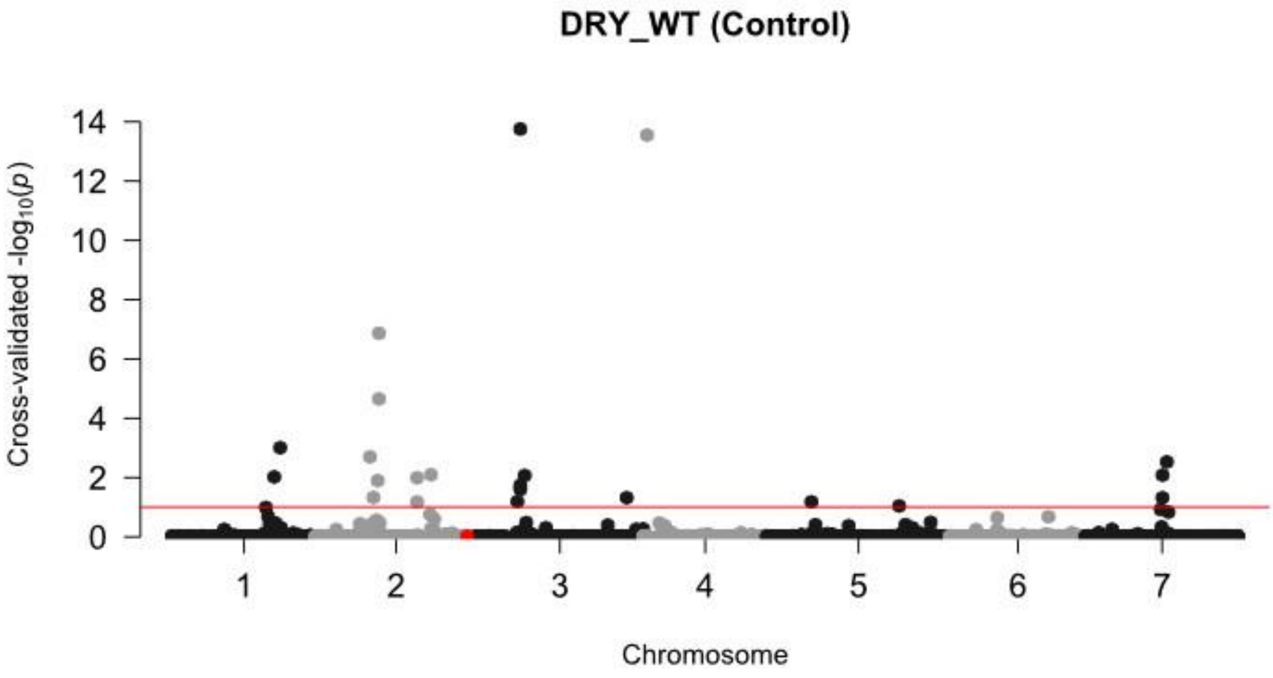

o) Manhattan plot for dry mass per m^2 under control conditions

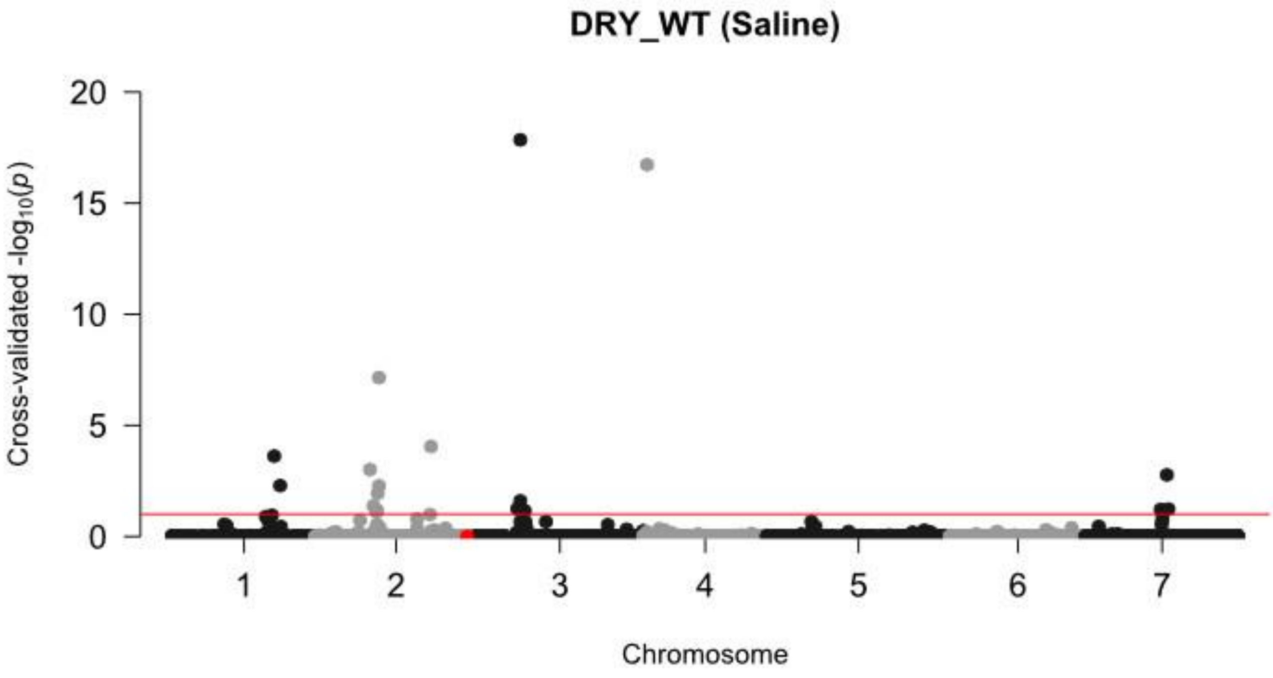

p) Manhattan plot for dry mass per m^2 under saline conditions

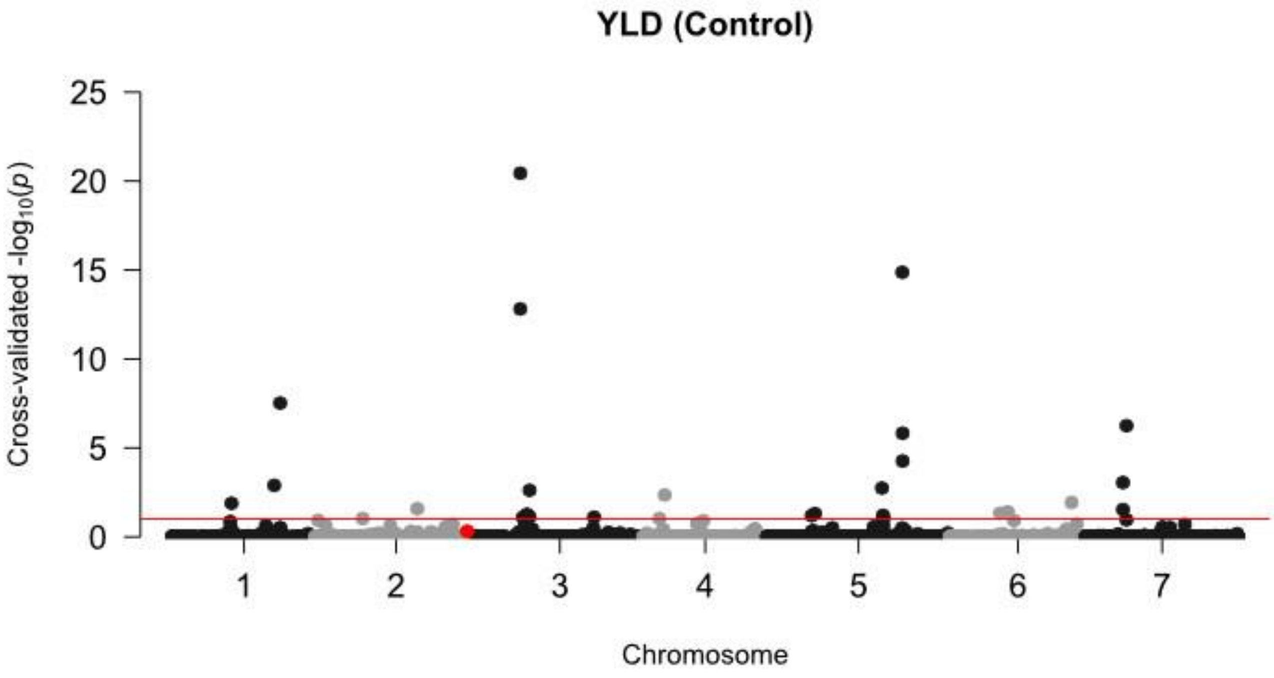

q) Manhattan plot for yield under control conditions

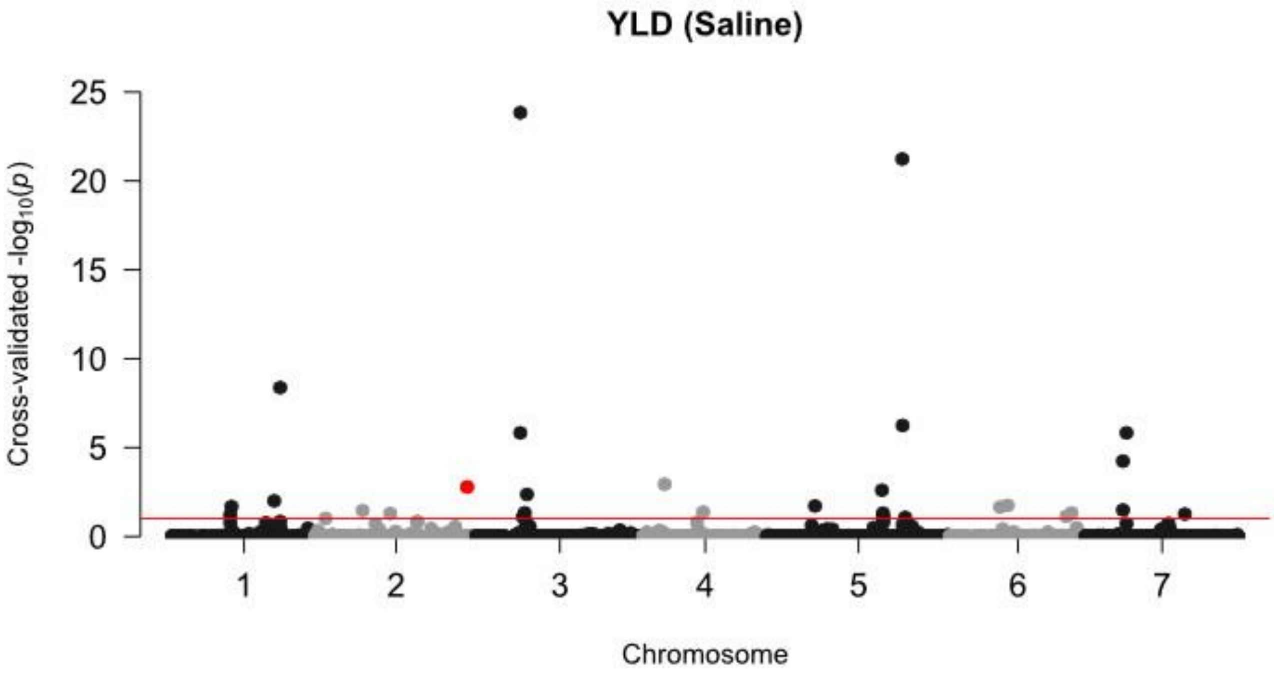

r) Manhattan plot for yield under saline conditions

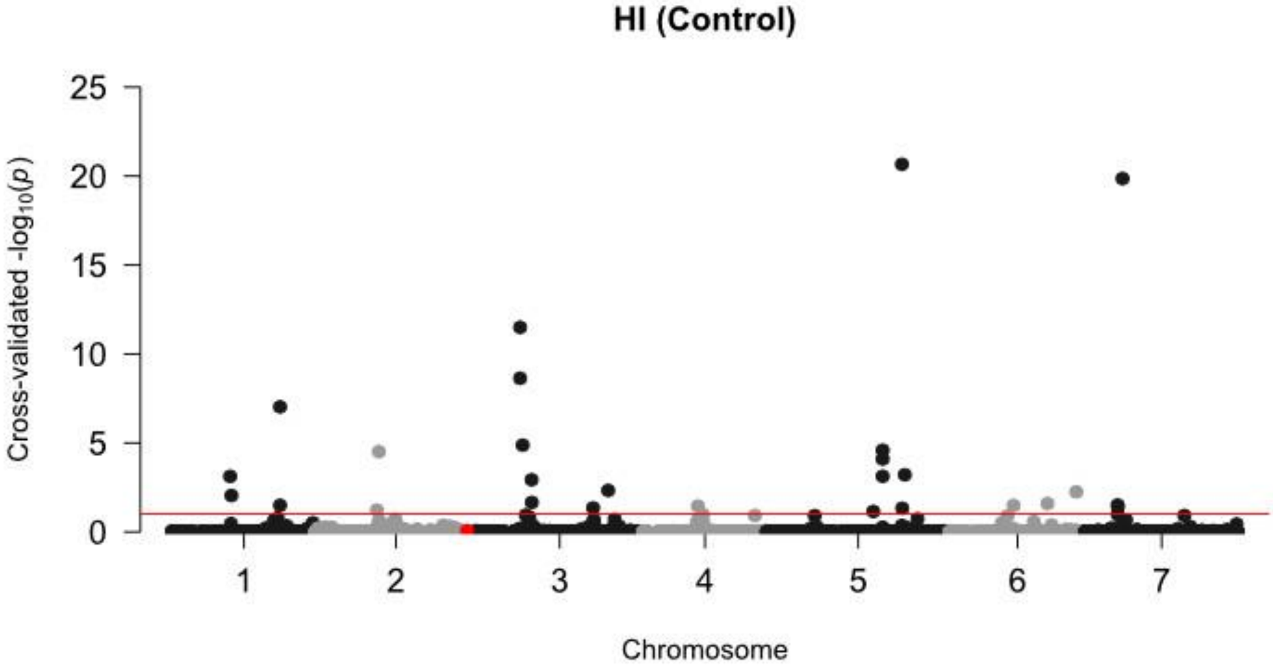

s) Manhattan plot for harvest index under control conditions

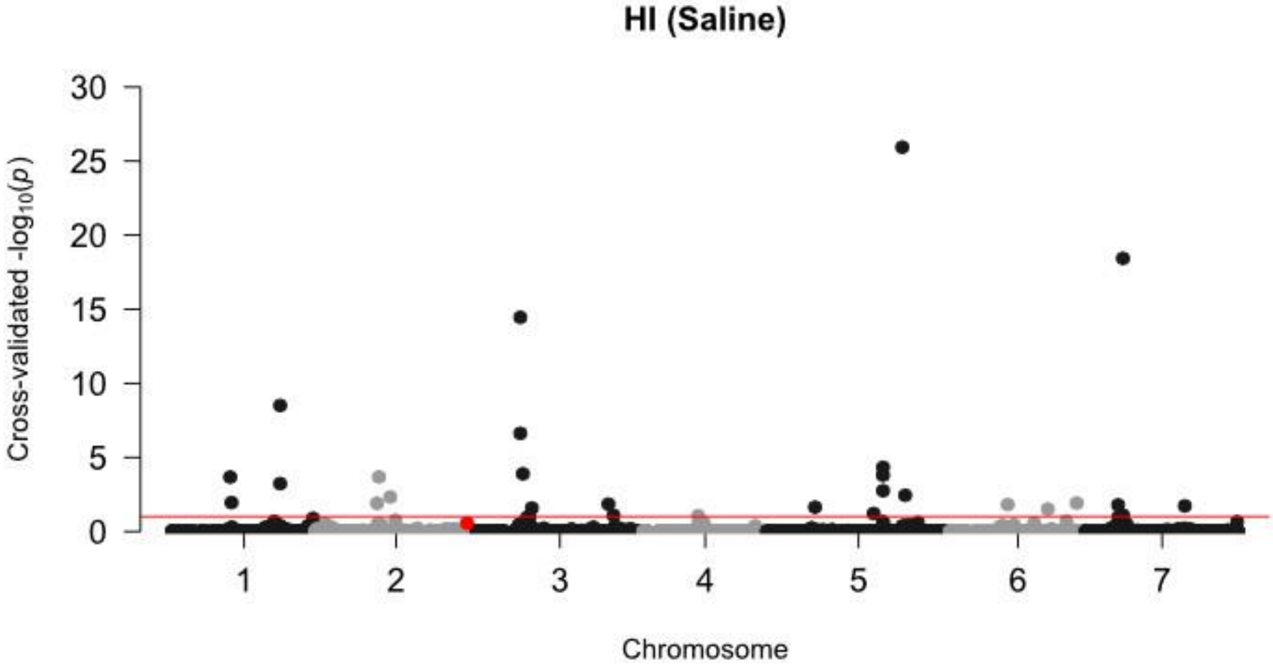

t) Manhattan plot for harvest index under saline conditions

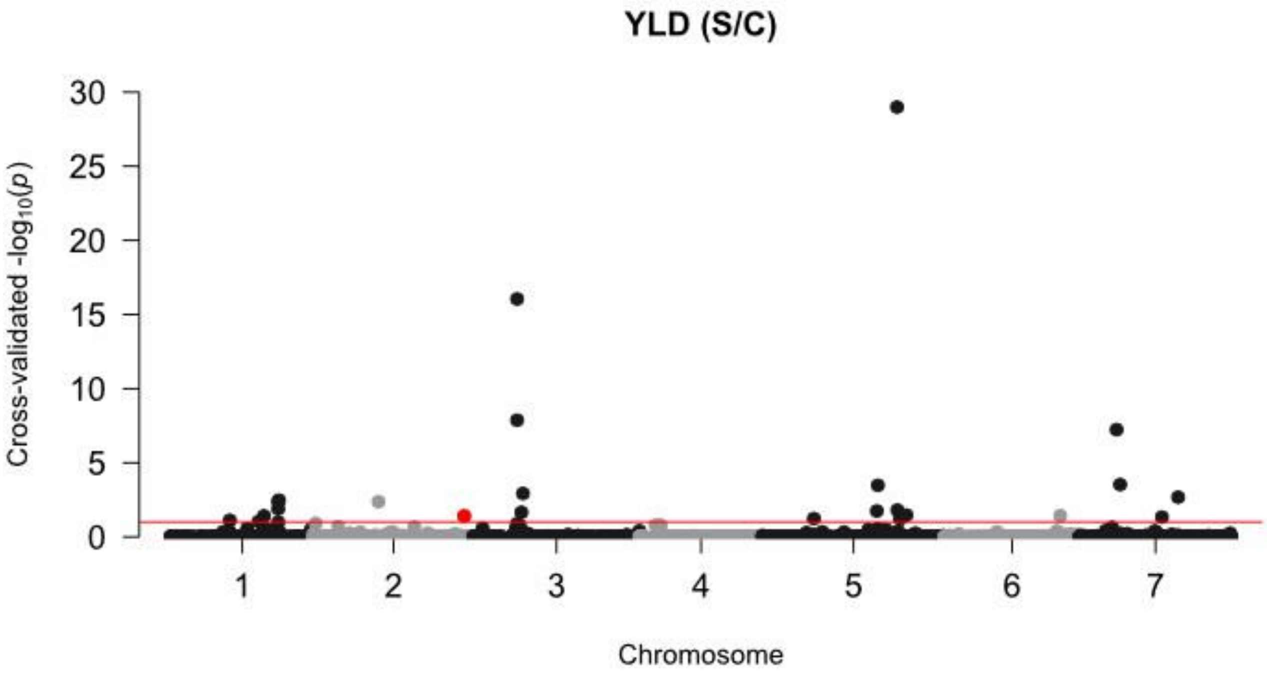

u) Manhattan plot for the stress tolerance index S/C

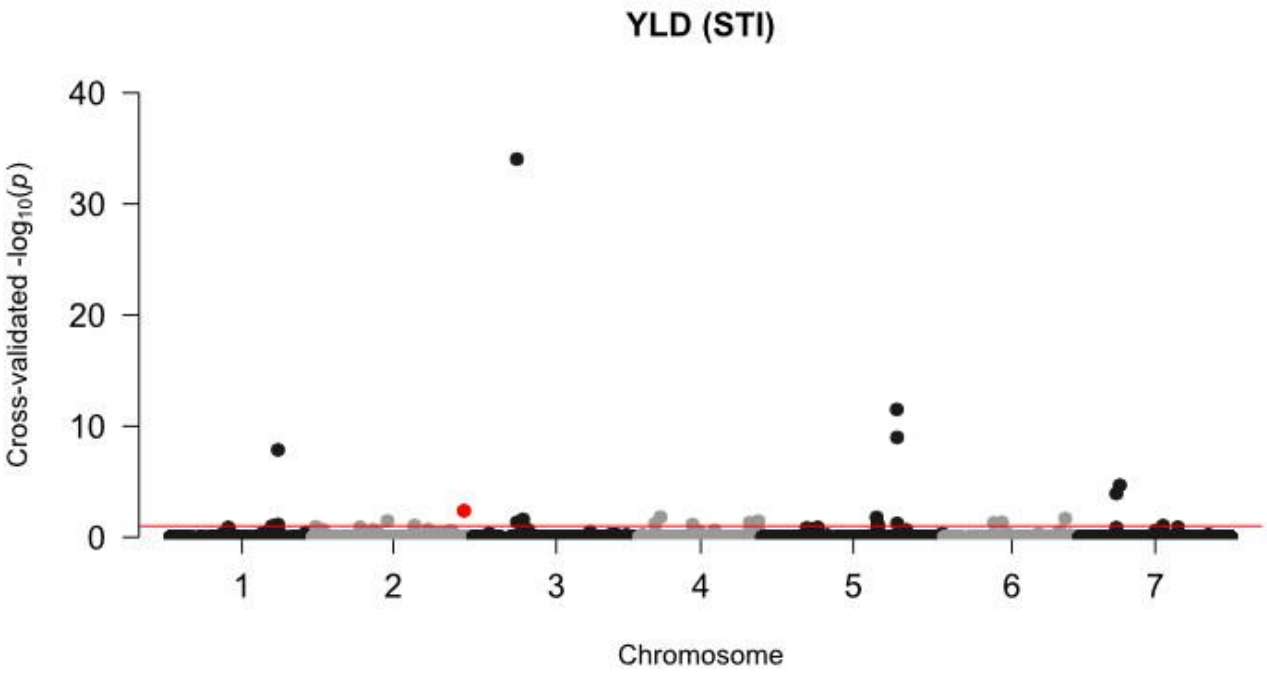

v) Manhattan plot for the stress tolerance index STI

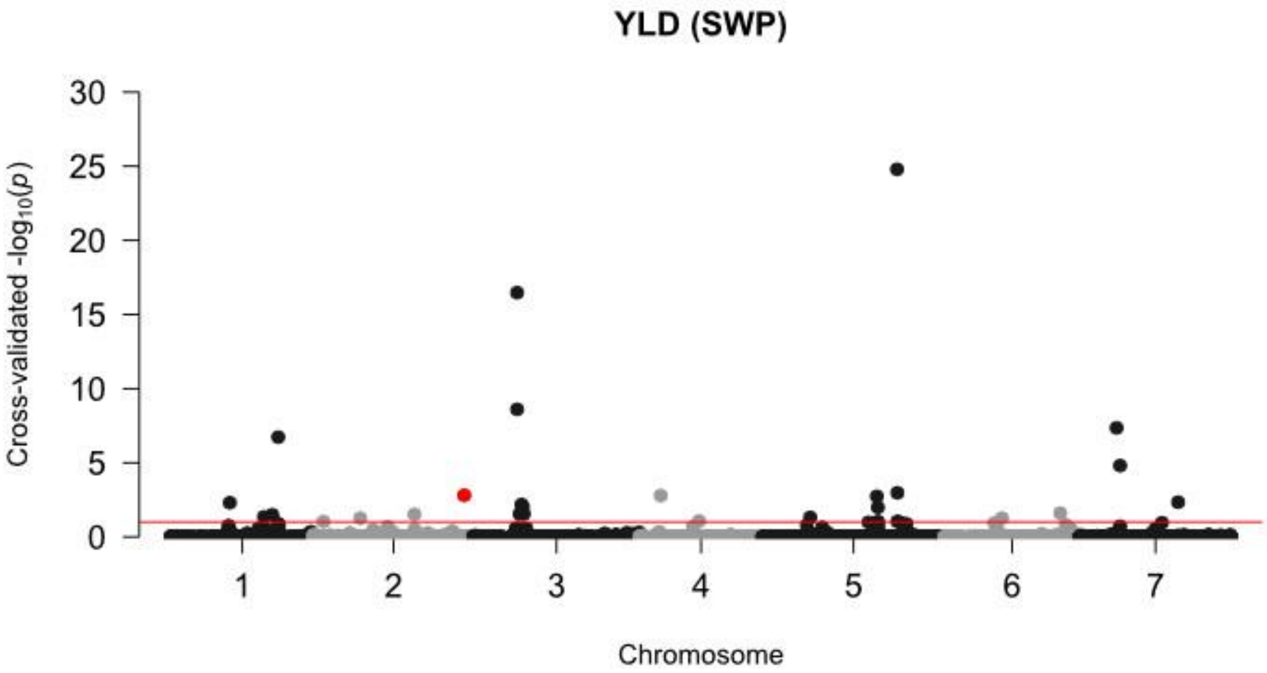

w) Manhattan plot for the stress tolerance index SWP

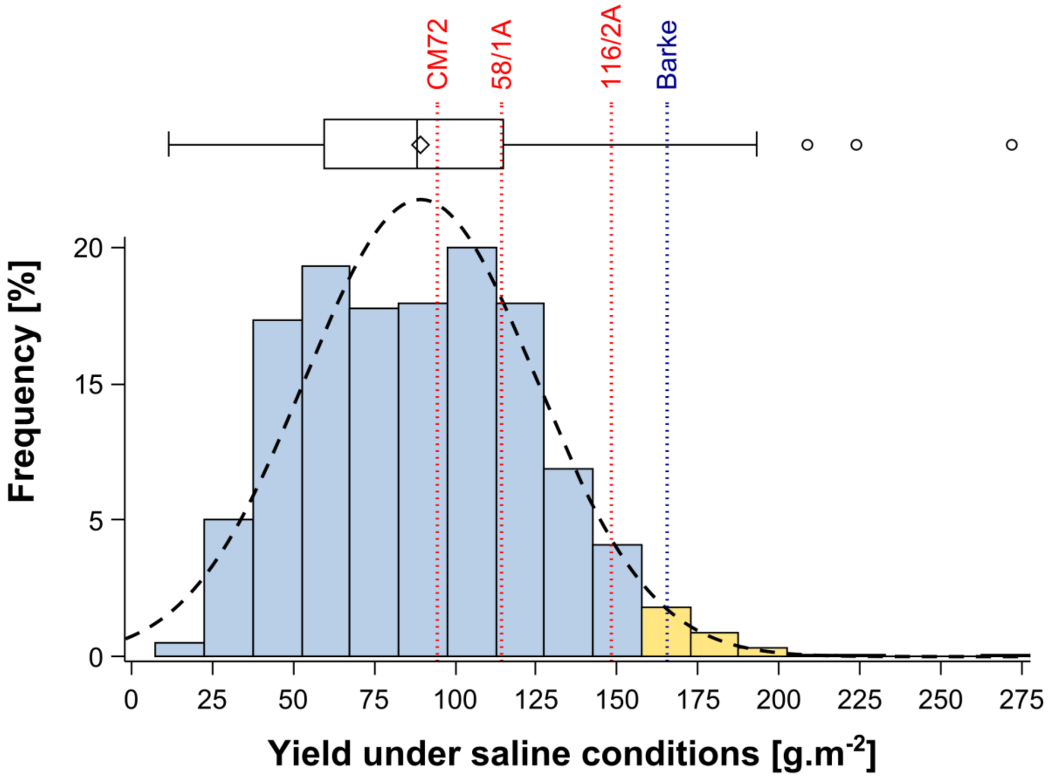

Supplementary Figure 3: Distribution of yield under saline conditions. This figure shows the frequency distribution of yield under saline conditions for HEB lines, Barke, and the three check lines (CM72, 58/1A, and 116/2A) used in the Dubai field trials. Barke, a German elite cultivar, has a high yield under Dubai field conditions and exhibits a high level of salinity tolerance (Table 1). Barke even outperforms the locally adapted check lines, which are considered to have a degree of salinity tolerance.

Supplementary Table 1: Number of lines studied per HEB family

Family number	Donor	Number of lines
1	HID_003	55
2	HID_004	49
3	HID_055	74
4	HID_062	40
5	HID_065	58
6	HID_069	57
7	HID_080	56
8	HID_099	54
9	HID_101	46
10	HID_102	58
11	HID_109	58
12	HID_114	68
13	HID_138	52
14	HID_140	60
15	HID_144	57
16	HID_219	58
17	HID_249	51
18	HID_270	22
19	HID_294	52
20	HID_295	51
21	HID_357	47
22	HID_358	42
23	HID_359	56
24	HID_380	58
25	HID_386	57
	Total	1,336

For an overview of the complete HEB-25 population, see Maurer, et al. [1].

Supplementary Table 2: Traits measured in the field under control and saline conditions

Trait	Abbreviation	Unit	Method of measurement
Flowering time	HEA	days	Number of days from sowing to BBCH scale code 55, when 50% of inflorescences emerged
Maturity time	MAT	days	Number of days from sowing to BBCH scale code 85, soft dough, where grain content is soft but dry and fingernail impression is not held
Ripening period	RIP	days	Time from HEA to MAT.
Plant height	HEI	cm	Measured prior to maturity as the distance from the ground to the plant tip including awns. The average of five representative plants was taken from the three middle rows of the plot
Ear number per plant	EAR	-	Measured at maturity as the average of five representative plants taken from the three middle rows of the plot
Grain number per ear	GPE	-	Measured at maturity as the average of five representative plants taken from the three middle rows of the plot
Thousand grain mass	TGW	g	Measured at harvest from the three middle rows of the plot based on a sample of 100 grains
Dry mass per m^2	DRY_WT	$g.m^{-2}$	Total above-ground dry mass, measured at harvest from the three middle rows of the plot
Yield (grain mass per m^2)	YLD	$g.m^{-2}$	Total grains, measured at harvest from the three middle rows of the plot
Harvest index	HI	-	Ratio of YLD to DRY_WT

Supplementary Table 3: Formulas to calculate salinity tolerance indices

Salinity tolerance index	Adapted from
$S/C = \frac{Y_S}{Y_C}$	Salt tolerance [3]
$STI = \frac{Y_C}{Y_{av}} \text{ x } \frac{Y_S}{Y_{av}}$	Stress tolerance index [4]
$SWP = \frac{Y_S}{\sqrt{Y_C}}$	Stress-weighted performance, this paper

Y_c represents the yield of a particular line under control conditions, Y_s the yield of a particular line under saline conditions, and Y_{av} the average yield for all the lines under control conditions.

Supplementary Table 4: Correlation of salinity tolerance indices for yield with other phenotypic traits. A heat map is used to color these correlations: blue indicates negative correlations, red indicates positive correlations, and the color intensity indicates the strength of the correlation (the darker the color the stronger the correlation). All correlations are significant ($p < 0.05$) except for the cells highlighted in yellow.

Trait/ stress tolerance index	Condition	S/C	STI	SWP
HEA	Control	-0.397	-0.362	-0.403
	Saline	-0.455	-0.384	-0.441
MAT	Control	-0.413	-0.37	-0.415
	Saline	-0.459	-0.381	-0.443
RIP	Control	0.248	0.237	0.258
	Saline	0.168	0.159	0.169
HEI	Control	0.00399	0.0334	0.0305
	Saline	0.149	0.0905	0.124
TGW	Control	0.138	0.185	0.173
	Saline	0.265	0.209	0.246
EAR	Control	0.586	0.615	0.672
	Saline	0.73	0.701	0.779
GPE	Control	0.714	0.72	0.792
	Saline	0.76	0.751	0.827
DRY_WT	Control	0.513	0.644	0.619
	Saline	0.494	0.603	0.584
YLD	Control	0.822	0.973	0.963
	Saline	0.888	0.975	0.989
HI	Control	0.8	0.883	0.912
	Saline	0.887	0.902	0.957
S/C			0.789	0.943
STI				0.934

Flowering time (HEA), maturity time (MAT), ripening period (RIP), plant height (HEI), thousand grain mass (TGW), ear number per plant (EAR), grain number per ear (GPE), dry mass per m^2 (DRY_WT), yield (YLD), and harvest index (HI). The stress tolerance indices S/C, STI and SWP are defined in Supplementary Table 3.

Supplementary Table 5: Weather data recorded at the field site at ICBA during the two years of field trials (2014 and 2015). Control plots for the first field trial were sown on December 10, 2013 and saline plots were sown on December 11, 2013. Control plots for the second field trial were sown on December 7, 2014 and saline plots on December 8, 2014.

Year	Month	Temperature (mean, in °C)	Maximum temperature (mean, in °C)	Minimum temperature (mean, in °C)	Maximum relative humidity (mean, in %)	Minimum relative humidity (mean, in %)	Precipitation (total, in mm)
2013	December	19.4	26.1	13.5	82.6	32.4	0.8
2014	January	16.8	23.2	11.1	85.0	36.5	11.6
2014	February	18.1	24.8	12.4	83.0	31.4	16.1
2014	March	21.4	28.7	15.3	82.3	26.7	12.0
2014	April	26.0	34.7	18.5	76.2	14.9	20.0
2014	May	29.1	37.8	21.0	69.2	13.9	2.6
2014	June	31.0	40.1	23.1	74.0	14.3	2.7
2014	July	32.9	40.6	26.2	73.1	18.1	1.4
2014	August	32.4	40.0	26.0	70.2	20.3	0.9
2014	September	32.0	40.6	25.0	77.1	17.5	1.1
2014	October	28.8	36.9	21.7	74.9	20	0
2014	November	23.0	30.1	16.3	75.5	26.2	0.2
2014	December	18.3	25.8	11.4	80.0	29.8	0.3
2015	January	16.8	24.4	10.0	86.2	28.3	25.2
2015	February	21.5	29.1	14.9	77.3	22.1	19.8
2015	March	20.2	28.0	13.3	76.7	19.2	0
2015	April	27.0	34.5	19.5	74.9	17.9	0
2015	May	32.6	40.5	24.7	57.8	13.9	0
2015	June	34.2	42.4	26.8	66.3	16	0
2015	July	35.9	44.4	28.8	63.7	12.6	0.8

Supplementary Table 6: Heritability in each test location (control 2013, control 2014, saline 2013, and saline 2014). Heritability (h^2) for the different traits measured shows that a substantial fraction of the phenotypic data is under genetic control, with h^2 for flowering time and yield being on the order of 80% and 30%, respectively.

	h^2 2013		h^2 2014	
	Condition		Condition	
Trait	Control	Saline	Control	Saline
HEA	0.807	0.646	0.786	0.821
MAT	0.672	0.653	0.680	0.792
RIP	0.262	0.183	0.688	0.317
HEI	0.609	0.573	0.863	0.624
TGW	0.564	0.607	0.675	0.608
EAR	0.625	0.552	0.685	0.154
GPE	0.391	0.230	0.644	0.147
DRY_WT	0.379	0.286	0.637	0.411
YLD	0.372	0.315	0.375	0.167
HI	0.537	0.471	0.477	0.327

Flowering time (HEA), maturity time (MAT), ripening period (RIP), plant height (HEI), thousand grain mass (TGW), ear number per plant (EAR), grain number per ear (GPE), dry mass per m^2 (DRY_WT), yield (YLD), and harvest index (HI)

Supplementary Table 7: R^2 of the training (R^2 train) and validation (R^2val) sets of all genome-wide association studies conducted for all traits (**a**) and stress tolerance indices (**b**).

a

	Condition			
	Control		Saline	
Trait	R^2train	R^2val	R^2train	R^2val
HEA	0.69	0.56	0.69	0.57
MAT	0.68	0.55	0.70	0.57
RIP	0.42	0.26	0.20	0.07
HEI	0.52	0.39	0.42	0.26
TGW	0.30	0.11	0.33	0.14
EAR	0.49	0.36	0.54	0.40
GPE	0.64	0.52	0.64	0.51
DRY_WT	0.40	0.24	0.40	0.24
YLD	0.59	0.44	0.59	0.45
HI	0.62	0.49	0.63	0.49

Flowering time (HEA), maturity time (MAT), ripening period (RIP), plant height (HEI), thousand grain mass (TGW), ear number per plant (EAR), grain number per ear (GPE), dry mass per m^2 (DRY_WT), yield (YLD), and harvest index (HI)

b

Stress tolerance indices for yield					
S/C		STI		SWP	
R^2train	R^2val	R^2train	R^2val	R^2train	R^2val
0.55	0.39	0.53	0.35	0.61	0.47

The stress tolerance indices S/C, STI, and SWP are defined in Supplementary Table 3.

Supplementary Dataset 1: Spatially adjusted data of the ten agronomic traits collected in the field under control and saline conditions.

(Excel file)

Supplementary Dataset 2: Significant single nucleotide polymorphisms of all genome-wide association studies conducted on all traits and stress-tolerance indices used in this study.

(Excel file)

Supplementary notes

Correlations between traits in the field

Flowering and maturity time are highly positively correlated and are both negatively correlated with ripening period (Table 2). In addition, flowering time and height are negatively correlated. Strong positive correlations are also found between yield, its components (ear number per plant and grain number per ear), and its related traits (dry mass per m^2 and harvest index). However, thousand grain mass, another component of yield, has the weakest correlation with yield compared with the other two components. A comparison of the current results with those of Schmalenbach, et al. [5](in which wild introgression lines were phenotyped under German field conditions) indicates that all the correlations have a similar direction except for thousand grain mass and grain number per ear. In Schmalenbach, et al. [5], thousand grain mass was the primary yield component (r=0.68) and negatively correlated with grain number per ear. In that same study, grain number per ear had a strong positive correlation with flowering time and a strong negative correlation with ear number per plant. Hence, we conclude that, under Dubai field conditions, grain number per ear seems to be a better determinant of yield than is thousand grain mass.

Under Dubai field conditions, we observe similar correlation trends under control and saline conditions except for correlations between flowering time and ripening period and between maturity time and ripening period, which become weaker under saline conditions compared with under control conditions, suggesting that plants adopt different strategies in response to high salinity.

Flowering loci control yield in the field

While earlier flowering has been related to higher yield under drought conditions [6], it is interesting to observe this relationship in irrigated field experiments, in which water is not a limiting factor. Earlier flowering in the current experiment is likely to be beneficial because it reduces plant exposure to higher temperatures that occur increasingly as the season progresses in Dubai (Supplementary Table 5).

Loci where genes controlling flowering time are located have significant effects on yield. Loci on chromosomes 4H (at 110-115 cM; cM positioning throughout the text follows Maurer, et al. [1]), 5H (at 105-110 and 125-130 cM) and 7H (at 30-35 cM), where *Vrn-H2* [7], *HvPRR95* [8], *Vrn-H1* [9] and *Vrn-H3* [10] are respectively located, all affect flowering. Moreover, the wild barley allele in all these loci, except *Vrn-H2*, also affects grain number per ear, yield, and harvest index under both control and saline conditions, as shown in the Circos plot (Figure 2).

We found that the *sdw1* locus (also known as *denso*) located on chromosome 3H (at 105-110 cM) [11] accelerates flowering and maturity time and extends the ripening period under both control and saline conditions. In addition, this locus increases plant height and reduces yield; but, importantly, its role in reducing yield is more prominent under control than under saline conditions. At the same time, *sdw1* plays a greater role in increasing thousand grain mass under saline than under control conditions. The combination of these effects leads to this gene playing a role in increasing the salinity tolerance of these plants. The wild allele at the *sdw1* locus is known to reduce both flowering and maturity time and to increase plant height and thousand grain mass compared with the cultivar allele [12]. In addition, a gibberellic acid (GA) GA-20 oxidase has been proposed as a candidate for the *sdw1* locus [11], and GA-to-ABA ratios have been shown to determine the differentiation of nucellar projections, an important step in the growth of

barley grains [13]. We suggest that the wild allele of *sdw1* might allow plants under saline stress to direct their potential more towards filling grain rather than increasing plant height.

Flowering and *Ppd* loci

The results of Maurer, et al. [1] showed that the *Ppd-H1* locus, located on chromosome 2H (at 20-25 cM) [14], was a major locus, with the wild barley allele promoting earlier flowering by 9.5 days. In contrast, under Dubai field conditions, the wild *Ppd-H1* allele delays flowering by 2 days. Furthermore, the frequency of *Ppd-H1* locus detection during cross validation is higher under control than under saline conditions, suggesting that genes other than *Ppd-H1* are likely to be involved in controlling flowering time under saline conditions. Interestingly, the *Ppd-H2* locus (also known as *HvFT3*) located on chromosome 1H (at 90-95 cM) [15] was not associated with flowering time in Maurer, et al. [1]; however, it was significantly associated in our field trials in Dubai. The wild barley allele at the *Ppd-H2* locus detected in our study also delays flowering (by approx. 2 days) and reduces yield and grain number per ear. It has been reported that *Ppd-H1* controls flowering time during long days, while *Ppd-H2* controls flowering time during short days [16]. Hence, the differences in detecting *Ppd-H1* and *Ppd-H2* are likely due to differences in day length, as this present study was performed in Dubai and the one by Maurer, et al. [1] in Germany.

Loci associated with yield

When studying yield in HEB-25, it is important to note that this trait is mainly influenced by brittleness of the rachis. The underlying genes, *Btr1* and *Btr2*, of this domestication-related trait have been recently cloned [17]. We clearly see this peak in the centromeric region of chromosome

3H (at 40-45 cM) as the major determinant of yield in our population, since no artificial selection was applied during population development in order to keep a maximum of genetic diversity.

A QTL on chromosome 4H (position 55-60 cM) is indicated by the genetic analyses, where the wild barley allele has a favorable effect on grain number per ears, yield, and the harvest index under both control and saline conditions. The wild allele increases yield by approximately 17 $g.m^{-2}$ and the harvest index by 0.03 under saline conditions, where the yield average is 89 $g.m^{-2}$ and harvest index is 0.21. The peak SNPs, BOPA2_12_10426 and BOPA1_5848-1413, lie within the genes coding an oxysterol-binding protein and a glycosyltransferase group 1 family protein, respectively. Further studies of these two candidate genes may unravel their importance in yield improvement.

References (supplementary material)

1. Maurer, A. *et al.* Modelling the genetic architecture of flowering time control in barley through nested association mapping. *Bmc Genomics* **16**(2015).
2. Holm, S. A Simple Sequentially Rejective Multiple Test Procedure. *Scandinavian Journal of Statistics* **6**, 65-70 (1979).
3. Munns, R. Comparative physiology of salt and water stress. *Plant, Cell & Environment* **25**, 239-250 (2002).
4. Fernandez, G.C.J. Effective selection criteria for assessing plant stress tolerance. in *Proceedings of the international symposium on adaptation of vegetable and other food crops in temperature and water stress* 257-270 (Taiwan, 1992).
5. Schmalenbach, I., Leon, J. & Pillen, K. Identification and verification of QTLs for agronomic traits using wild barley introgression lines. *Theoretical and Applied Genetics* **118**, 483-497 (2009).
6. Bose, B. *Developments in Physiology, Biochemistry and Molecular Biology of Plants*, (New India Publishing Agency, 2008).
7. Yan, L. *et al.* The wheat *VRN2* gene is a flowering repressor down-regulated by vernalization. *Science* **303**, 1640-1644 (2004).
8. Cockram, J. *et al.* Genome dynamics explain the evolution of flowering time CCT domain gene families in the Poaceae. *PLoS One* **7**, e45307 (2012).
9. Cockram, J., Horsnell, R., Soh, E.H., Norris, C. & O'Sullivan, D.M. Molecular and phenotypic characterization of the alternative seasonal growth habit and flowering time in barley (*Hordeum vulgare* ssp *vulgare* L.). *Molecular Breeding* **35**(2015).
10. Yan, L. *et al.* The wheat and barley vernalization gene *VRN3* is an orthologue of *FT*. *Proceedings of the National Academy of Sciences of the United States of America* **103**, 19581-19586 (2006).
11. Jia, Q. *et al.* GA-20 oxidase as a candidate for the semidwarf gene *sdw1/denso* in barley. *Funct Integr Genomics* **9**, 255-62 (2009).
12. Kuczynska, A. *et al.* Effects of the semi-dwarfing *sdw1/denso* gene in barley. *J Appl Genet* **54**, 381-90 (2013).
13. Weier, D. *et al.* Gibberellin-to-abscisic acid balances govern development and differentiation of the nucellar projection of barley grains. *J Exp Bot* **65**, 5291-304 (2014).
14. Turner, A., Beales, J., Faure, S., Dunford, R.P. & Laurie, D.A. The pseudo-response regulator Ppd-H1 provides adaptation to photoperiod in barley. *Science* **310**, 1031-4 (2005).
15. Kikuchi, R., Kawahigashi, H., Ando, T., Tonooka, T. & Handa, H. Molecular and functional characterization of *PEBP* genes in barley reveal the diversification of their roles in flowering. *Plant Physiology* **149**, 1341-1353 (2009).
16. Laurie, D.A., Pratchett, N., Snape, J.W. & Bezant, J.H. RFLP mapping of five major genes and eight quantitative trait loci controlling flowering time in a winter x spring barley (*Hordeum vulgare* L.) cross. *Genome* **38**, 575-85 (1995).
17. Azhaguvela, P., Vidya-Saraswathia, D. & Komatsudaa, T. High-resolution linkage mapping for the non-brittle rachis locus *btr1* in cultivated × wild barley *(Hordeum vulgare)*. *Plant Science* **170**, 1087–1094 (2006).
